# Antibacterial Efficacy of Ethanol Extracts from Edible *Rumex madaio* Root and Application Potential for Eliminating *Staphylococcus aureus* and *Vibrio cholerae* in Aquatic Products for Green Food Preservation

**DOI:** 10.3390/foods14203479

**Published:** 2025-10-12

**Authors:** Huanhuan Fan, Yue Liu, Enyu Tian, Yaping Wang, Shunlin Ren, Bailin Li, Huajun Zheng, Lanming Chen

**Affiliations:** 1 College of Food Science and Technology, Shanghai Ocean University, Shanghai 201306, China; 2 Department of Internal Medicine, McGuire VA Medical Centre, Virginia Commonwealth University, Richmond, VA 23298, USAshunlin.ren@vcuhealth.org (S.R.); 3 Shanghai Institute for Biomedical and Pharmaceutical Technologies, Shanghai 200237, China

**Keywords:** *Rumex madaio*, antibacterial activity, antibacterial mechanism, green food preservation, natural product, food safety

## Abstract

Edible and medicinal plants provide a treasure trove of natural phytochemicals for mining the next generation of green food preservatives. Herein, we evaluated antibacterial activities of 55–95% ethanol extracts from the edible rhizome of *Rumex madaio* (*Rm*EEs). The 75% ethanol extract displayed the strongest antibacterial activity, and its purified fraction 2 (*Rm*EE-F2) blocked the proliferation of common pathogens *Staphylococcus aureus* and *Vibrio cholerae*, with minimum inhibitory concentrations (MICs) of 391 μg/mL. *Rm*EE-F2 (1 × MIC) altered the bacterial cell surface biophysical parameters and impaired cell structure, resulting in intracellular nucleic acid and protein leakage. It manifested bacteriostatic rates of 88.21–91.17% against *S. aureus* and *V*. *cholerae* in spiked fish (*Carassius auratus*) and shrimp (*Penaeus vannamei*) during storage at 4 °C for 24 h. Meanwhile, *Rm*EE-F2 effectively rendered the pH rising and reduced lipid oxidation and protein degradation of *C. auratus* and *P. vannamei* meat samples at 4 °C for 6 days. Additionally, *Rm*EE-F2 (< 781 µg/mL) showed non-cytotoxicity to human colon Caco-2, liver HepG-2, and lung A549 cell lines, and rescued *V. cholerae* and *S. aureus*-infected Caco-2 cellcells with enhanced viability of 14.31–16.60% (1 × MIC). Comparative transcriptomic analysis revealed down-regulated protein synthesis, cell wall and cell membrane synthesis, and or DNA replication and repair in the tested bacteria triggered by *Rm*EE-F2. The major antibacterial compounds in *Rm*EE-F2 included melibiose (9.86%), 3-(N, N-dimethylaminomethyl) indole (7.12%), and citric acid (6.07%). Overall, this study underscores the promising potential of *Rm*EE-F2 for aquatic product green preservation.

## 1. Introduction

Foodborne pathogens pose a major risk to food safety and human health [1]. According to the World Health Organization (WHO), 420,000 people die each year due to consuming contaminated food by foodborne and waterborne pathogens [2]. Antibiotics have been used to control infectious diseases for more than 70 years. Nevertheless, the overuse and misuse of antibiotics escalates selection pressure, leading to the emergence and transmission of antibiotic resistance (AR) [1,3]. It is estimated that tens of millions of people would die from AR annually by 2050, if no new antibiotic drugs are available [4]. Hence, the discovery of new bacteriostatic agents and or alternative strategies is imperative for harnessing AR foodborne pathogens [5].

Phytochemicals in edible and medicinal plants provide a vast source for mining green antibacterial substances [6,7,8,9]. *Rumex madaio* Makino. is such a perennial herbaceous plant that serves as a traditional medicine in Southern Africa, America, China, India, and Turkey for a long history [10]. For instance, the earliest medicinal efficacy of *R. madaio* was recorded in “Divine Farme’s Materia Medica” in 1828 in China [10]. So far, extensive efforts have been expended on bioactivities of extracts from *Rumex* species, such as anti-inflammation, anti-tumor, anti-diabetes, anti-hemorrhage, and anti-diarrhea [11,12]. Nevertheless, current literature on their antibacterial efficacy remains limited. Recently, Liu et al. [13] first reported that the methanol extract of *R. madaio* (designated as *Rm*ME) contained antibacterial components against several common species of foodborne pathogens.

In comparison with methanol, ethanol is a food-grade and environmentally friendly solvent. Bioactive compounds (e.g., phenols and flavonoids) can be dissolved in a wide range of ethanol concentrations [14]. Moreover, ethanol can reduce cost-associated energy consumption [15]. Therefore, in this study, we aimed to: (1) investigate extraction efficiency of different ethanol concentrations (55–95%) for extracting antibacterial components in the rhizome of *R. madaio*; (2) analyze antibacterial activity and mode of the purified fraction 2 of 75% ethanol extract from *R. madaio* (*Rm*EE-F2); and (3) evaluate application potential of *Rm*EE-F2 for eliminating *Staphylococcus aureus* and *Vibrio cholerae* contamination in fish (*Carassius auratus*) and shrimp (*Penaeus vannamei*) during the low temperature (4 °C) storage. This study unlocks the potential of *Rm*EE-F2 as a natural green preservative for aquatic product storage.

## 2. Results and Discussion

### 2.1. Antibacterial Effects of RmEEs Extracted with Different Concentrations of Ethanol

In this study, the fresh rhizome sample of *R. madaio* was freeze-dried at −80 °C for 48 h, and its water loss was 78.84%. Extraction ratios of the sample were 25.57%, 33.8%, and 31.10% when 95%, 75%, and 55% ethanol were used as solvents, respectively.

As shown in Table S1, all the *Rm*EEs could inhibit 7 of the 9 tested bacterial species, but showed various DIZs (17.00–8.00 mm). In contrast, the growth of *E. coli* ATCC 25922 and *Enterobacter cloacae* ATCC 13047 was not suppressed by *Rm*EEs. Intrinsic (e.g., efflux pumps) or structural (e.g., microcapsules or peripheral pili) factors might account for the selective antibacterial activity among different Gram-negative strains.

The most pronounced inhibitory activity was manifested by *Rm*EE (95% E or 75% E) against Gram-positive *S. aureus* ATCC 25923, followed by Gram-negative *V. cholerae* GIM 1.449, showing DIZ values of 17.00 ± 0.50 and 11.50 ± 0.32 mm, respectively, which had a similar antibacterial effect as the positive control gentamicin (CN, 10 μg/mL) (DIZs: 18 ± 0.30, and 23 ± 0.20). Correspondingly, minimum inhibitory concentrations (MICs) of *Rm*EE (95% E or 75% E) against *S. aureus* ATCC 25923 and *V. cholerae* GIM 1.449 were 98 μg/mL and 391 μg/mL, respectively (Table S1).

The previous study has indicated that *Rm*ME also inhibited *S. aureus* ATCC 25923 and *V. cholerae* GIM 1.449, with DIZ values of 8.10 ± 0.29 mm and 10.50 ± 0.41 mm, and MIC values of 512 μg/mL and 128 μg/mL, respectively [13]. These differences between *Rm*ME and *Rm*EE suggested solvent-dependent extraction efficiency of antibacterial components and complexity of phytochemical compounds in the rhizome of *R. madaio.*

Given that the raw material cost of 75% E is lower than that of 95% E, *Rm*EE (75% E) with the highest extraction rate was chosen for further analyses in this study.

### 2.2. Purification of the RmEE (75% E)

**Figure 1:**
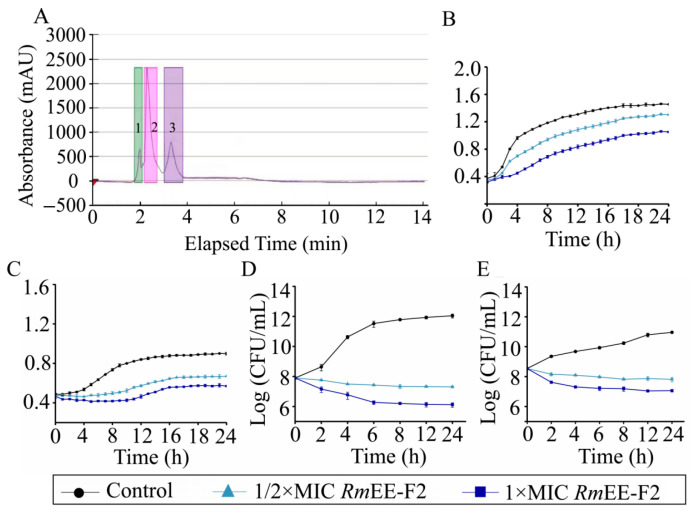
Bacteriostatic effects of the ethanol extracts from the rhizome of *R. madaio*. (**A**) The Prep-HPLC purification of the *Rm*EE (75% E). The peaks marked in green, pink and purple represented the purified fractions 1, 2 and 3, respectively. (**B**,**C**) Growth curves of *V. cholerae* GIM 1.449 and *S. aureus* ATCC 25923 incubated in the TSB supplemented with *Rm*EE-F2 (1 × MIC, or 1/2 × MIC) at 37 °C, respectively. (**D**,**E**) Time-killing curves of *V. cholerae* GIM 1.449 and *S. aureus* ATCC 25923 incubated in the TSB supplemented with *Rm*EE-F2 (1 × MIC, or 1/2 × MIC) at 37 °C, respectively. Control, without *Rm*EE-F2.

As shown in Figure 1A, we further purified *Rm*EE (75% E) through Pre-HPLC analysis, and observed three distinct fractions (designated as *Rm*EE-F1 to *Rm*EE-F3), scanning at 211 nm for 14 min. *Rm*EE-F2, occurring during 2.2 to 2.8 min, manifested the strongest inhibition effect on *S. aureus* ATCC 25923, followed by *V. cholerae* GIM 1.449, with MIC values of 391 μg/mL and 391 μg/mL, and DIZ values of 11.50 ± 0.50 and 10.50 ± 0.35 mm, respectively. Additionally, *Rm*EE-F1 and *Rm*EE-F3 showed relatively weaker inhibition activity (Table S2). In comparison with *Rm*EEs, the decreased DIZ values of *Rm*EE-F2 may be attributed to certain antibacterial components with lower abundance lost during the fractionation process, and their synergistic antimicrobial effects lost after the fractionation. Similar observation has been reported in previous reports [13,16].

Given that the study of isolating and characterizing single photochemicals paves the way for developing highly targeted antibacterial agents, and that the purified *Rm*EE-F2 showed a lighter color and smell than those of crude extract *Rm*EEs, more suitable for developing natural food preservatives in aquatic products, the *Rm*EE-F2 was chosen for further analysis in this study.

### 2.3. RmEE-F2 Inhibited Growth and Changed Cell Biophysical Parameters of the Tested Strains

As shown in Figure 1B, compared with the control group (the maximum OD_600_ = 1.46), the growth of *S. aureus* ATCC 25923 was significantly inhibited after being treated with *Rm*EE-F2 (1 × MIC, 391 μg/mL), with the maximum biomass (OD_600_ = 1.03) decreased by 0.30-fold (*p* < 0.05). Similarly, the 1/2 × MIC of *Rm*EE-F2 decreased the bacterial maximum biomass (OD_600_ = 1.31) by 0.12-fold (*p* < 0.05). The *Rm*EE-F2 concentration-dependent decrease (0.37-fold and 0.26-fold) in the growth was also observed against *V. cholerae* GIM 1.449, compared with the control group (*p* < 0.05) (Figure 1C).

To confirm these results, we also determined the time-killing curves of the tested strains. As shown in Figure 1D, compared with the control group, viable *S. aureus* ATCC 25923 counts reduced by 1.49, 3.86, 5.26, 5.59, 5.79 and 5.93 Log reduction (colony forming unit (CFU)/mL) after being treated with *Rm*EE-F2 (1 × MIC, 391 μg/mL) for 2 h, 4 h, 6 h, 8 h, 12 h and 24 h, respectively; and reduced by 0.89 to 4.74 Log reduction (CFU/mL) with 1/2 × MIC of *Rm*EE-F2 for 2 h to 24 h. Similarly, as shown in Figure 1E, compared with the control group, viable *V. cholerae* GIM 1.449 counts also reduced by 1.73 to 3.90 and 1.20 to 3.15 Log reduction (CFU/mL) after being treated with 1 × MIC and 1/2 × MIC of *Rm*EE-F2 (1 × MIC) for 2 to 24 h, respectively.

**Figure 2:**
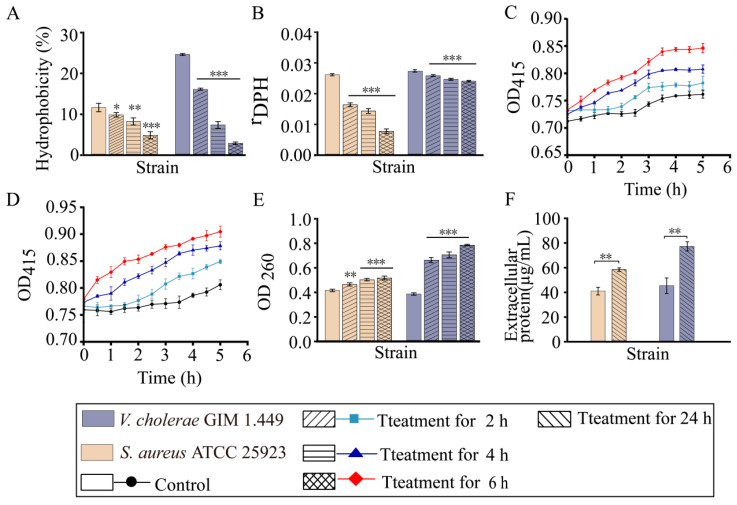
The effects of *Rm*EE-F2 (1 × MIC) on CSH (**A**), CMF (**B**), ICMP (**C**,**D**), nucleic acid (**E**), and protein (**F**) exudation of *S. aureus* ATCC 25923 and *V. cholerae* GIM 1.449. The results were expressed as mean ± standard deviation (SD). * *p* < 0.05; ** *p* < 0.01; and *** *p* < 0.001.

Bacterial cell surface hydrophobicity (CSH) is one of the key cell biophysical parameters and affects cell-cell and cell-surface interactions [17]. As shown in Figure 2A, in comparison with the control groups, the CSH of *S. aureus* ATCC 25923 reduced by 1.18-fold (*p* < 0.05), 1.42-fold (*p* < 0.01), and 2.47-fold (*p* < 0.001) after treated with *Rm*EE-F2 (1 × MIC) for 2 h, 4 h, and 6 h, respectively (*p* < 0.001); and the more pronounced decrease was observed in the CSH of *V. cholerae* GIM 1.449 by 1.54-fold, 3.43-fold, and 9.17-fold (*p* < 0.001), respectively.

Cell membrane fluidity (CMF) influences cell membrane protein and lipid interaction [18]. We observed that the CMF of *S. aureus* ATCC 25923 significantly increased by 1.62-fold, 1.86-fold, and 3.71-fold, after being treated with *Rm*EE-F2 (1 × MIC) for 2 h, 4 h, and 6 h, respectively (*p* < 0.001) (Figure 2B). Likewise, the CMF of *V. cholerae* GIM 1.449 also increased by 1.05-fold to 1.14-fold (*p* < 0.001). Higher DPH values pointed to weaker CMF [16].

The bacterial cytoplasmic membrane is a selective permeability barrier against drugs entering the cell [19]. Most antibacterial drugs functioning at intracellular processes must pass through the bacterial cell envelope [20]. As shown in Figure 2C,D, both the tested strains showed an increasing trend in inner cell membrane permeability (ICMP) (1.11-fold and 1.13-fold), after being treated with *Rm*EE-F2 (1 × MIC) for 6 h (*p* < 0.05).

The previous study by Liu et al. [13] indicated that the purified fraction 1 from *Rm*ME (designated as *Rm*ME-CC1) significantly increased CSH and CMF of *V. parahaemolyticus* ATCC 17802. In this study, collectively, our results showed that *Rm*EE-F2 can significantly reduce CSH, but increase CMF and ICMP of both Gram-positive *S. aureus* ATCC 25923 and Gram-negative *V. cholerae* GIM 1.449 strains. These changes might have influenced the bacterial colonization, changed membrane protein function, and caused intracellular substance leakage, leading to bacterial death.

### 2.4. RmEE-F2 Led to Nucleic Acid and Protein Exudation and Damaged Cell Structure of the Tested Strains

As shown in Figure 2E, the amount of nucleic acids exuded from *S. aureus* ATCC 25923 was significantly on the rise by 1.12-fold to 1.24-fold after being treated with *Rm*EE-F2 (1 × MIC) for 2–6 h (*p* < 0.01). The similarly rising was also observed in *V. cholerae* GIM 1.449 (1.73-fold to 2.05-fold, *p* < 0.001). Meanwhile, the extracellular protein contents of *S. aureus* ATCC 25923 and *V. cholerae* GIM 1.449 significantly increased by 1.43-fold and 1.71-fold, respectively, after being treated with *Rm*EE-F2 for 24 h (*p* < 0.01) (Figure 2F). Collectively, both nucleic acid and protein significantly exuded from the two tested strains, suggesting possible cell damage caused by *Rm*EE-F2. Therefore, we further observed the bacterial cell structure change by the scanning electron microscope (SEM) analysis.

**Figure 3:**
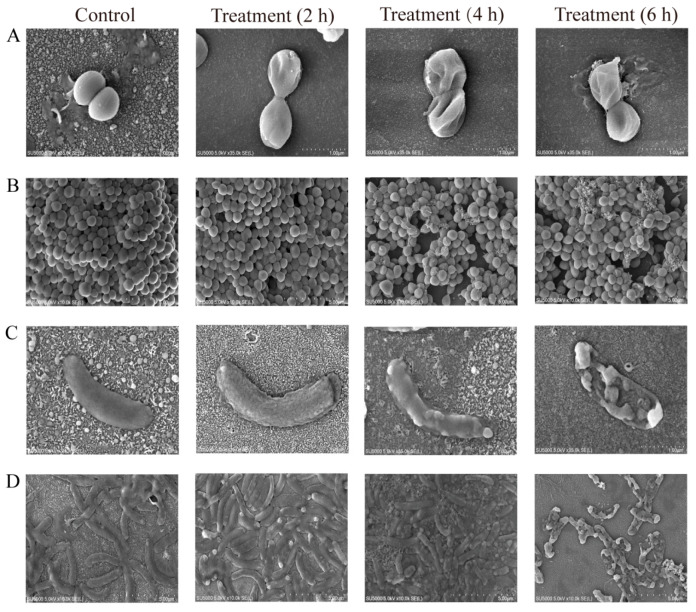
The SEM analysis of the cell surface structure of *S. aureus* ATCC 25923 and *V. cholerae* GIM 1.449 before and after treatment by *Rm*EE-F2 for different times. *S. aureus* ATCC 25923 (**A**,**B**) and *V. cholerae* GIM 1.449 (**C**,**D**) were treated with *Rm*EE-F2 (1 × MIC) for 2 h, 4 h, and 6 h, respectively. The magnification of colonies and the bacterial population was × 35,000 and × 10,000, respectively.

As shown in Figure 3A,B, compared with the control group that showed full clear cell structure, the depressed and dented cell surface of *S. aureus* ATCC 25923 was shown after treated with *Rm*EE-F2 (1 × MIC) for 2 h; the more severely assaulted appearance occurred after 4 h of the treatment; and *S. aureus* ATCC 25923 cells were broken with the extrusion of certain cellular contents after 6 h of the treatment. Similarly, the obvious wrinkling on the cell surface was observed in the *V. cholerae* GIM 1.449 treatment group for 2 h; severe damage occurred for 4 h; and a large amount of the broken cells appeared for 6 h (Figure 3C,D).

These results indicated that the cell structure of Gram-positive and Gram-negative bacteria can be severely damaged by *Rm*EE-F2 (1 × MIC), showing a treatment time-dependent blockage mode.

### 2.5. RmEE-F2 Disturbed Metabolic Pathways in the Tested Strains

On the basis of the above findings, we determined transcriptomes of *S. aureus* ATCC 25923 and *V. cholerae* GIM 1.449 before and after the *Rm*EE-F2 (1 × MIC) treatment, to explore molecular mechanisms of the antibacterial activities of *Rm*EE-F2.

#### 2.5.1. Major Disturbed Metabolic Pathways in *S. aureus* ATCC 25923 Triggered by RmEE-F2

**Figure 4:**
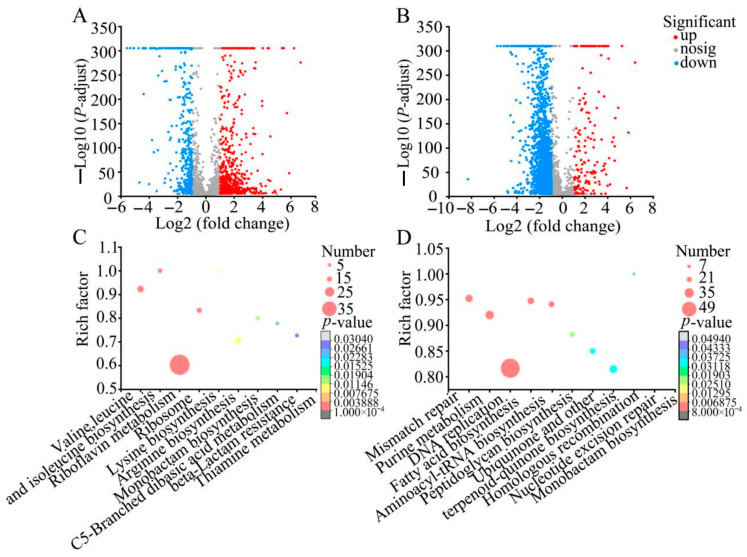
The significantly altered metabolic pathways in *S. aureus* ATCC 25923 (**A**,**C**) and *V. cholerae* GIM 1.449 (**B**,**D**) after being treated with *Rm*EE-F2 (1 × MIC) for 6 h. (**A**,**B**) Volcano plots of the DEGs. (**C**,**D**) The altered metabolic pathways.

Approximately 41.79% (959/2295) of genes in *S. aureus* ATCC 25923 were significantly changed after being treated with *Rm*EE-F2 (1 × MIC) for 6 h, compared with the control group (*p* < 0.05). Of these, 389 differentially expressed genes (DEGs) were down-regulated (fold change (FC) ≤ 0.5), and 570 DEGs were up-regulated (FC ≥ 2.0) (Figure 4A). The DEGs were enriched in 9 metabolic pathways, e.g., arginine biosynthesis, leucine and isoleucine biosynthesis, lysine biosynthesis, ribosome, valine, and beta-lactam resistance (*p* < 0.05) (Figure 4C, Table S3).

For example, in the ribosome pathway, approximately 9 DEGs were significantly down-regulated in *S. aureus* ATCC 25923 (0.216-fold to 0.489-fold) (*p* < 0.05). Ribosome dysfunction perturbs translational fidelity [21]. For example, the expression of 50S ribosomal protein L7/L12 (*rplL*, *PQQ26_02565*) was significantly hindered at the transcription level (0.240-fold) (*p* < 0.05). This protein catalyzes peptide bond formation in the peptidyltransferase center and directs the nascent polypeptide chain through the exit tunnel [22]. The 50S ribosomal protein L14 (*rplN*, *PQQ26_11285*) was also significantly repressed (0.216-fold) (*p* < 0.05). This protein has been demonstrated to have antibacterial and anti-biofilm activities against multidrug-resistant *S. aureus* in vitro and in vivo [23].

In the lysine and arginine biosynthesis, approximately 11 DEGs were significantly down-regulated (0.094-fold to 0.492-fold) (*p* < 0.05). For instance, the expression of aspartate-semialdehyde dehydrogenase (*PQQ26_06670*), a key enzyme in microbial amino acid biosynthesis and cell wall formation [24], was significantly repressed (0.492-fold) (*p* < 0.05). Remarkably, argininosuccinate lyase (*argH*, *PQQ26_04235*), a terminal enzyme in arginine biosynthesis [25], was strongly down-regulated (0.094-fold, *p* < 0.05). L-arginine affects stress response and virulence factor expression in pathogenic bacteria [26]. Arginine and its metabolites also serve as energy substrates for multiple pathogens [27].

In the β-lactam resistance, approximately 12 DEGs were significantly down-regulated in *S. aureus* ATCC 25923 (0.323-fold to 0.495-fold) (*p* < 0.05). The expression of DEG (*PQQ26_05490*) encoding a penicillin-binding protein (PBP) was reduced at the transcription level (0.329-fold) (*p* < 0.05). PBPs, the principal targets for β-lactam antibiotics, are crucial in the construction of the cell wall and maintenance of cell shape [28]. A reduction in the synthesis of PBPs mitigates the bacterial resistance to β-lactam drugs.

AMP is a widely used β-lactam antibiotic drug [29]. In this study, our results indicated that *S. aureus* ATCC 25923 was resistant to AMP with a MIC value of 64 µg/mL. However, after being treated with *Rm*EE-F2 (1 × MIC) for 6 h, the MIC value of AMP was 8 µg/mL, significantly decreased by 8-fold, compared to the untreated control (*p* < 0.05). This result verified the transcriptomic data and demonstrated that *Rm*EE-F2 has a synergistic effect and mitigates *S. aureus* ATCC 25923 resistance to β-lactam drugs (e.g., AMP).

In contrast, in the riboflavin metabolism, the expression of 9 DEGs was significantly up-regulated in *S. aureus* ATCC 25923 (2.016-fold to 10.789-fold) (*p* < 0.05). Flavoenzymes fuel cellular energy and govern growth and viability via metabolic redox networks [30]. For example, the riboflavin synthase (*ribE*, *PQQ26_08905*), which participates in the riboflavin biosynthesis at the final step, was highly increased (10.757-fold, *p* < 0.05) [31], which may offset the growth impairment triggered by *Rm*EE-F2.

Liu et al. [13] reported that *Rm*ME affected motility, energy metabolism, and substance transport in Gram-positive *Bacillus cereus*, thus inhibiting its growth. Herein, our results indicated that *Rm*EE-F2 mainly inhibited protein synthesis and β-lactam resistance, resulting in impaired growth, the elimination of drug resistance, and even the death of *S. aureus* ATCC 25923.

#### 2.5.2. Major Disturbed Metabolic Pathways in *V. cholerae* GIM 1.449 Triggered by RmEE-F2

Approximately 61.3% (2301/3753) of genes in *V. cholerae* GIM 1.449 were significantly changed after being treated with *Rm*EE-F2 (1 × MIC) for 6 h (*p* < 0.05). Among these, 2213 DEGs were down-regulated (FC ≤ 0.5), and 88 DEGs were up-regulated (FC ≥ 2.0) (Figure 4B). The DEGs were enriched in 10 significantly altered metabolic pathways, e.g., DNA replication, homologous recombination, aminoacyl-tRNA biosynthesis, mismatch repair (MMR), peptidoglycan biosynthesis, nucleotide excision repair (NER), and fatty acid biosynthesis (*p* < 0.05) (Figure 4D; Table S4).

For example, in the DNA replication and NER, approximately 23 DEGs were significantly inhibited in *V. cholerae* GIM 1.449 (0.195-fold to 0.468-fold) (*p* < 0.05). NER is the most versatile DNA repair pathway to eliminate various bulky DNA lesions [32]. For instance, the ribonuclease HII (*rnhB*, *GTH07_03155*), a key enzyme that deletes misincorporated ribonucleoside monophosphates from genomic DNA [33], was significantly repressed (0.374-fold, *p* < 0.05).

In the MMR, approximately 20 DEGs were significantly hindered (0.183-fold to 0.469-fold) (*p* < 0.05). The MMR maintains genomic integrity [34]. For instance, the DNA MMR protein MutS (*mutS*, *GTH07_11350*) and DNA MMR endonuclease MutL (*mutL*, *GTH07_12235*) were significantly down-regulated (0.257-fold and 0.316-fold), respectively (*p* < 0.05), whose inactivation can increase mutation rates and frequencies in bacteria [35]. The expression of core DNA polymerase (*dnaE*, *GTH07_03160*; *dnaQ*, *GTH07_03215*), sliding clamp (*dnaN*, *GTH07_00010*), and multi-protein clamp loader (*dnaX*, *GTH07_08890*; *holA*, *GTH07_09345*) was all curtailed (0.312-fold to 0.375-fold) (*p* < 0.05). They are crucial components of DNA polymerase III required for bacterial replication and growth [36]. The down-regulated MMR may have resulted in the deficient ability of *V. cholerae* GIM 1.449 to repair DNA damage, leading to cell death.

In the fatty acid biosynthesis, approximately 23 DEGs were significantly down-regulated in *V. cholerae* GIM 1.449 (0.090-fold to 0.428-fold) (*p* < 0.05). This pathway is essential for forming phospholipids, lipopolysaccharides/lipooligosaccharides, and Gram-negative bacterial envelope lipoproteins [37]. For instance, β-ketoacyl-ACP synthase II (*fabF*, *GTH07_04290*) was hindered (0.166-fold) (*p* < 0.05). This enzyme catalyzes fatty acid elongation in fatty acid synthase type II (FAS-II), which is essential for bacterial cell membrane construction [38].

In the peptidoglycan biosynthesis, approximately 19 DEGs were significantly down-regulated in *V. cholerae* GIM 1.449 (0.166-fold to 0.431-fold) (*p* < 0.05). Peptidoglycan acts as a vital structural component in the bacterial cell wall [39]. MurA (*GTH07_01875*) catalyzes the first step of peptidoglycan synthesis; MurC (*GTH07_02455*) and MurE (*GTH07_02425*) ligases catalyze sequential condensation of L-Ala, meso-diaminopimelic acid/L-Lys, D-Glu, and D-Ala in peptidoglycan assembly [40]; MraY (*GTH07_02435*) catalyzes the transfer of phospho-N-acetylmuramoyl-pentapeptide moiety to undecaprenyl phosphate lipid carrier; and MurG (*GTH07_02450*) catalyzes the last step of peptidoglycan synthesis [41]. Expression of all these enzymes was significantly hindered (0.321-fold to 0.432-fold) (*p* < 0.05) in *V. cholerae* GIM 1.449. In addition, the DEGs encoding penicillin-binding proteins (*mrcB*, *GTH07_11015*; *GTH07_01290*; *mrdA*, *GTH07_09360*; *GTH07_02420*) were repressed as well (0.231-fold to 0.388-fold) (*p* < 0.05), which are required for cross-linking peptidoglycan stem peptides. These proteins are recognized as potential targets for developing successful antibiotics, due to their central roles in bacterial cell wall synthesis [42].

Collectively, *Rm*EE-F2 mainly inhibited DNA replication and repair, cell wall and cell membrane synthesis, and protein synthesis, leading to cell damage and death of *V. cholerae* GIM 1.449.

Additionally, the reverse-transcription quantitative PCR (RT-qPCR) validation of ten key DEGs showed consistent expression trends with the transcriptome data (Figure S1).

### 2.6. RmEE-F2 Effectively Killed the Tested Strains in the Spiked Fish and Shrimp During the Low-Temperature Preservation

China is the leading consumer, producer, and exporter of aquatic products worldwide [43]. Along with the promoted production, there is also an increasingly rising demand for green preservatives in aquatic products [44]. In this study, we assessed the inhibitory efficacy of *Rm*EE-F2 on *S. aureus* ATCC 25923 and *V. cholerae* GIM 1.449 in artificially spiked *C. auratus* and *P. vannamei* during low-temperature (4 °C) storage.

#### 2.6.1. *Rm*EE-F2 Effectively Killed the Tested Strains in the *C. auratus* Sample

**Figure 5:**
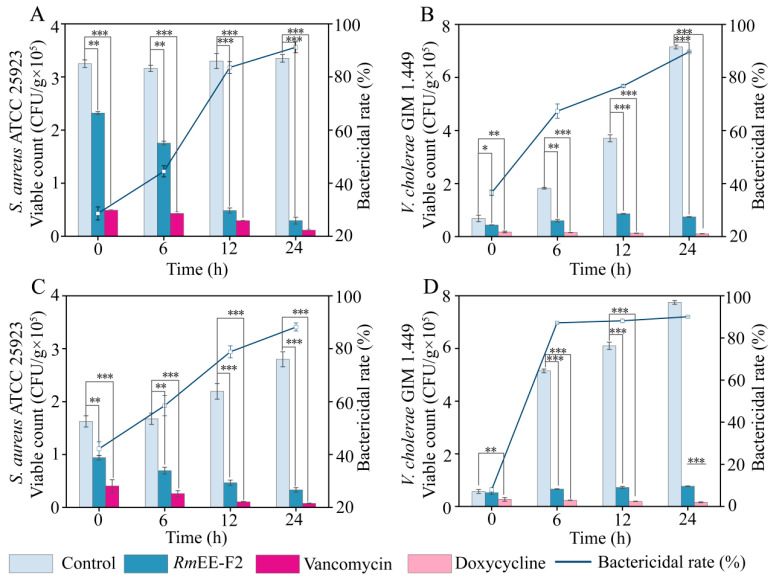
The inhibition activity of *Rm*EE-F2 (1 × MIC) on the spiked *C. auratus* (**A**,**B**) and *P. vannamei* (**C**,**D**) samples. (**A**,**C**) Spiked with *S. aureus* ATCC 25923. (**B**,**D**) Spiked with *V. cholerae* GIM 1.449. * *p* < 0.05, ** *p* < 0.01, and *** *p* < 0.001.

As presented in Figure 5A, viable counts of *S. aureus* ATCC 25923 in the *C. auratus* sample were significantly decreased by 0.26 Log CFU/g (*p* < 0.01), 0.83 Log CFU/g (*p* < 0.001) and 1.01 Log CFU/g (*p* < 0.001), after treated with *Rm*EE-F2 (39.1 μg/g) at 4 °C for 6 h, 12 h and 24 h, respectively, in comparison with the control group. Correspondingly, the bacteriostatic rates against *S. aureus* ATCC 25923 were 44.4%, 83.57%, and 91.17%, respectively.

Similarly, the viable counts of *V. cholerae* GIM 1.449 in the *C. auratus* sample were also significantly reduced by 0.49 Log CFU/g (*p* < 0.01), 0.63 Log CFU/g (*p* < 0.001), and 0.99 Log CFU/g (*p* < 0.001), after the 6 h, 12 h, and 24 h treatment, respectively. Correspondingly, the bacteriostatic rates against *V. cholerae* GIM 1.449 were 67.32%, 76.79%, and 89.65%, respectively (Figure 5B).

These results indicated that the bacteriostatic rates of *Rm*EE-F2 against *S. aureus* ATCC 25923 and *V. cholerae* GIM 1.449 in *C. auratus* displayed a treatment time-dependent increase, reaching the highest (91.17% and 89.65%) after 24 h treatment at 4 °C, and showing similar effects as the positive controls.

#### 2.6.2. RmEE-F2 Effectively Killed the Tested Strains in the *P. vannamei* Sample

As presented in Figure 5C, the viable count of *S. aureus* ATCC 25923 in the *P. vannamei* sample was significantly reduced by 0.38 Log CFU/g (*p* < 0.01), 0.67 Log CFU/g (*p* < 0.001), and 0.93 Log CFU/g (*p* < 0.001), after treated with *Rm*EE-F2 (39.1 μg/g) at 4 °C for 6 h, 12 h, and 24 h, respectively. Correspondingly, the bacteriostatic rates were 58.51%, 78.82%, and 88.21%, respectively.

Likewise, the viable count of *V. cholerae* GIM 1.449 in the *P. vannamei* sample manifested a decrease by 0.89 Log CFU/g, 0.93 Log CFU/g, and 1.0 Log CFU/g after the 6 h, 12 h, and 24 h treatment, respectively (*p* < 0.001). Correspondingly, the bacteriostatic rates were 87.18%, 88.11%, and 90.01%, respectively (Figure 5D).

To the best of our knowledge, only a few food preservatives have been approved for use in the food industry, such as nisin, tea polyphenols, and sorbic acid. Of these, the allowable maximum addition limits in aquatic products, as well as cooked and processed aquatic products (e.g., fish, crustaceans and shellfish) are as follows: nisin is 0.5 g/kg; tea polyphenols are 0.3 g/kg; and sorbic acid is 1.0 g/kg (National Food Safety Standard for Use of Food Additives, GB 2760–2024, China) [45]. For example, a study by Wijnker et al. [46] showed that nisin (50 μg/mL) decreased the outgrowth of *Clostridium sporogenes* spores by 1 Log reduction (90%) in natural sausage casings at ambient temperature for 8 days. Essential oils (EOs) have been widely used in the food industry. Djenane [47] reported that Lemon EO (100 µL/mL) reduced *S. aureus* in sardines by 3.80 log10 cfu/g (88.37% reduction) when stored at 8 °C for 7 days, as compared to the control. Recently, Ahmad et al. [48] reported that the ethanol extract of *Annona muricata* L. reduced the growth of *Aeromonas* spp., *Enterobacter* spp., and *V. parahaemolyticus* in Pacific white shrimp.

Herein, we found that *Rm*EE-F2 markedly eliminated *S. aureus* ATCC 25923 and *V. cholerae* GIM 1.449 in the *P. vannamei* sample, reaching the highest bacteriostatic rates (88.21% and 90.01%) after the 24 h treatment, showing similar effects as the positive controls. As a potential green food preserver, *Rm*EE is still at its early development stage, and no official standard is available so far. In this study, *Rm*EE-F2 was added at an effective antibacterial concentration of 0.0391 g/kg in *C. auratus* and *P. vannamei* samples, much lower than those of the above food preservers, aligning with the safe exposure levels. Notably, the different food matrices (*C. auratus* and *P. vannamei*) did not significantly influence the antibacterial effect of *Rm*EE-F2. Nevertheless, more aquatic product matrices should be evaluated in future research.

### 2.7. RmEE-F2 Effectively Preserved Quality and Sensory of the Fish and Shrimp Meat Samples at the Low-Temperature Storage

**Figure 6:**
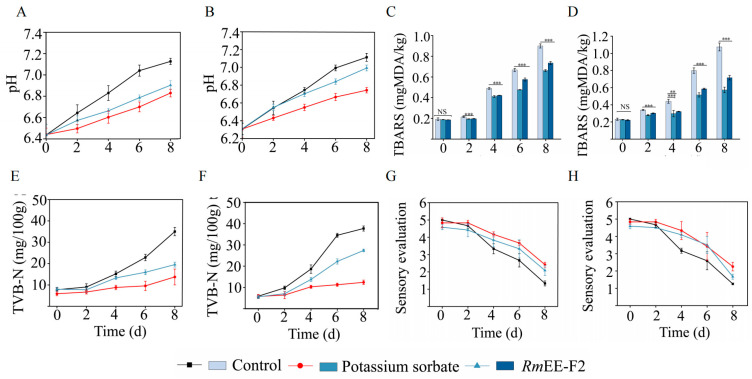
The effects of *Rm*EE-F2 on pH (**A**,**B**), TBARS (**C**,**D**), TVB-N (**E**,**F**), and sensory (**G**,**H**) of the *P. vannamei* and *C. auratus* meat samples during storage at 4 °C for 8 days. (**A**,**C**,**E**,**G**) and (**B**,**D**,**F**,**H**) represented the *P. vannamei* and *C. auratus* meat samples, respectively. MDA represented 1,1,3,3-tetramethoxypropane-derived malondialdehyde. NS represented no significance. ** *p* < 0.01; and *** *p* < 0.001.

The pH value is considered a key indicator of the freshness of aquatic products [49]. As presented in Figure 6A,B, we observed that the pH values of all the samples showed an upward tendency during the 4 °C storage for 8 days. This increase may be attributed to the accumulation of volatile bases (e.g., biogenic amines, ammonia, and trimethylamine) through protein hydrolysis by microorganisms and or endogenous enzymes in aquatic products [50]. However, the pH values of the control groups increased the fastest for both *C. auratus* and *P. vannamei* samples and reached pH 7.0 on day 6, compared with the treatment groups, indicating the fastest decay. In contrast, the groups treated with *Rm*EE-F2 manifested a slower increase in pH (<pH 7.0 on day 8), indicating its effective preservation effects against the decaying process.

The thiobarbituric acid reactive substances (TBARS) are secondary oxidation products (e.g., aldehydes and ketones) of lipids, leading to undesirable rancid odor in food [51]. As presented in Figure 6C,D, in the control groups, the TBARS values rose rapidly, reaching 1.07 ± 0.05 mg 1,1,3,3-tetramethoxypropane-derived malondialdehyde (MDA) /kg (*C. auratus* sample) and 0.90 ± 0.02 mg MDA/kg (*P. vannamei* sample) during the 4 °C storage for 8 days. However, after being treated with *Rm*EE-F2, the TBARS levels of the *C. auratus* and *P. vannamei* meat samples were 0.72 ± 0.03 and 0.74 ± 0.01 mg MDA/kg on day 8, respectively, significantly lower than those in the control groups during the storage (*p* < 0.001). These results indicated that *Rm*EE-F2 effectively reduced lipid oxidation and meat spoilage, thereby extending the shelf life of *C. auratus* and *P. vannamei* meat samples during storage.

The total volatile basic-nitrogen (TVB-N) value is also an important indicator of aquatic product spoilage [52], due to oxidative deamination of amino acids caused by microorganisms and or endogenous enzymes [53]. As presented in Figure 6E,F, in the control groups, the TVB-N levels also showed a significantly upward tendency, particularly after being stored at 4 °C for 4 days in comparison with the treatment groups, indicating that the quality of the samples deteriorated and spoilage became more severe. In contrast, the TVB-N values of *P. vannamei* and *C. auratus* meat samples treated by *Rm*EE-F2 were 19.52 ± 1.14 and 27.38 ± 0.60 mg/100 g on day 8, which were notably lower than those of the control groups (*p* < 0.001). Based on the China National Standard (GB 2733-2015) [54], the TVB-N threshold for aquatic products is established at < 30 mg/100 g. The results of this study indicated that the *Rm*EE-F2 treatment effectively delayed the spoilage process, thereby better preserving the freshness of the *C. auratus* and *P. vannamei* meat samples. The superior performance of the *Rm*EE-F2 treatment may be attributed to its ability to hinder protease activity and block protein degradation in the samples, thereby effectively extending the shelf life of the samples.

As shown in Figure 6G,H, the sensory score of the control groups was unacceptable when stored for 6 days at 4 °C (2.67 for *P. vannamei* and 2.58 for *C. auratus*). In contrast, the scores of the treatment groups were acceptable (>3.50 score) on day 6, although the samples showed a slight sour taste, the overall colour changed, and the meat texture softened.

Collectively, *Rm*EE-F2 can effectively render the pH rising and reduce lipid oxidation and protein degradation of the *C. auratus* and *P. vannamei* meat samples during the low-temperature preservation, thereby preventing the meat spoilage, and extending the shelf life of the aquatic products.

### 2.8. RmEE-F2 Showed No Cytotoxicity on Human Caco-2, HepG-2 and A549 Cells and Rescued Caco-2 cells Infested by the Tested Strains

**Figure 7:**
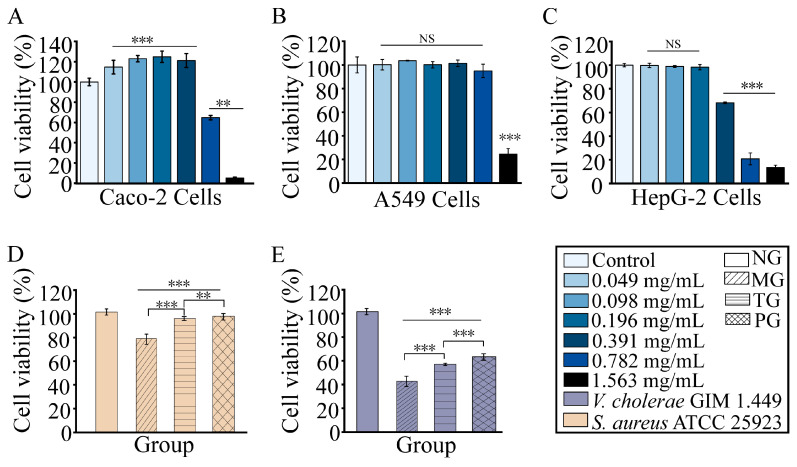
The cytotoxicity of *Rm*EE-F2. (**A**–**C**) Human colon Caco-2 (**A**), lung A549 (**B**), and liver HepG-2 (**C**) cell lines were treated with different concentrations of *Rm*EE-F2, respectively. (**D**,**E**) Viability of Caco-2 cells infected by *S. aureus* ATCC 25923 (**D**) and *V. cholerae* GIM 1.449 (**E**) before and after treatment with *Rm*EE-F2 (1 × MIC), respectively. NS represented no significance. ** *p* < 0.01, and *** *p* < 0.001.

We further evaluated the biosafety of bioactive ingredients in *Rm*EE-F2 through in vitro cell mode experiments. As presented in Figure 7A–C, compared with the control group, after treated with *Rm*EE-F2 (0.049–0.391 mg/mL) at 37 °C for 24 h, the viability of human colon adenocarcinoma Caco-2 cells had a significant increase (14.62–24.83%, *p* < 0.001), whereas no significant change in the viability of human lung adenocarcinoma A549 and hepatocellular carcinoma HepG-2 cells (*p* > 0.05), except that the viability of HepG-2 cells reduced to 68.19% after treated with *Rm*EE-F2 (391 µg/mL). The higher concentration (1.563 mg/mL) of *Rm*EE-F2 significantly inhibited all the tested cells.

According to the International Organization for Standardization (ISO, 10993-5) [55], percentages of cell viability above 80%, within 80–60%, 60–40% and below 40% are considered as non-cytotoxicity, weak, moderate, and strong cytotoxicity, respectively. In this study, *Rm*EE-F2 (391 µg/mL) reduced HepG-2 cell viability to 68.19%, indicating weak toxicity to HepG-2 cells. These results provided a valuable window of safe concentrations for the subsequent development and utilization of *Rm*EE-F2.

Based on the above findings, we further set up the Caco-2 cell model infected by *V. cholerae* GIM 1.449 and *S. aureus* ATCC 25923, and evaluated the viability of Caco-2 cells before and after treating the tested strains with *Rm*EE-F2 (1 × MIC). As shown in Figure 7D,E, compared to the negative control group (NG), the viability of Caco-2 cells in the model group (MG) was significantly decreased by 21.26% and 57.30% (*p* < 0.001), after being infected by *S. aureus* and *V. cholerae,* respectively.

After treatment with *Rm*EE-F2 (1 × MIC), compared to the MG cells, the viability of Caco-2 cells in the treatment group (TG) was significantly elevated by 16.60% (infected by *S. aureus* ATCC 25923) and 14.3% (infected by *V. cholerae* GIM 1.449) (*p* < 0.001), showing a similar effect as the positive control group (PG). These results indicated that *Rm*EE-F2 can significantly reduce the negative impact of the test strains on the Caco-2 cell, likely caused by the blocked growth and assaulted cell structure of the tested strains after being treated by *Rm*EE-F2.

### 2.9. Potential Antibacterial Compounds in RmEE-F2

We further identified potential antibacterial compounds in *Rm*EE-F2, and the results uncovered the highest relative abundance of melibiose (C_12_H_22_O_11_, 9.86%), followed by 3-(N, N-dimethylaminomethyl) indole (3-Nid, C_11_H_14_N_2_, 7.12%), citric acid (C_6_H_8_O_7_, 6.01%), 3-methylbutanamine (C_5_H_13_N, 5.18%), and otonecine (C_9_H_15_NO_3_, 4.42%) (Table 1, Table S5). Studies have revealed that the absorbance wavelength of citric acid and otonecine were 210 nm and 200 nm, respectively [56,57], which provided additional evidence to verify the results yielded from the preparative-high performance liquid chromatography (Prep-HPLC) analysis.

Melibiose, a reducing disaccharide containing galactose and glucose via an α-1,6 glycosidic bond, exhibited therapeutic potential for atopic dermatitis [58]. The 3-Nid, an indole alkaloid, has been reported for anti-inflammatory, anti-tumor, anti-bacterial, anti-viral, and anti-fungal activities [59]. Citric acid in aqueous extracts of *Lonicera caerulea* L. showed antibacterial activity against *B. subtilis* [60].

The previous study by Liu et al. has shown that in *Rm*ME-CC1, amino acids and derivatives accounted for the highest abundance (37.42%), followed by alkaloids (26.48%) and carbohydrates (26.5%) [13]. In this study, in *Rm*EE-F2, alkaloids had the highest percentage (20.60%), followed by carbohydrates (20.23%), and flavonoids (18.52%), indicating the effectiveness of ethanol solvent for extracting antibacterial compounds in the rhizome of *R. madaio*.

The main limitation of this study was that the in vivo antibacterial activity of *Rm*EE-F2 has not been demonstrated by animal mode experiments yet. More studies still need to be performed in future research. Additionally, *R. madai* is widely cultured as an edible and medicinal plant in China. The output per mu of its fresh root can reach up to 800 kg, and the price of dried root of *R. madai* is acceptable. These benefit the potential applications of *R. madai* in the food industry. However, the stability and long-term biosafety should be further investigated in future studies.

## 3. Materials and Methods

### 3.1. Bacterial Strains and Culture Conditions

*Vibrio* strains used in this study were routinely incubated in sterile Tryptic Soy Broth (TSB, pH 8.0–8.5, 3% NaCl) or Marine 2216 media, including *V. cholerae* GIM 1.449, *Vibrio metschnikovii* ATCC 700040 and *Vibrio parahaemolyticus* ATCC 17802, while *non-Vibrio* strains, including *Aeromonas hydrophila* ATCC 35654, *B. cereus* Y1, *E. cloacae* ATCC 13047, *E. coli* ATCC 25922, *Shigella dysenteriae* CMCC 51252 and *S. aureus* ATCC 25923, in the TSB (pH 7.0–7.2, 1% NaCl) or Luria-Bertani (LB, pH 7.0–7.2) at 37 °C, until mid-logarithmic growth phase (mid-LGP, OD_600_ = 0.6–0.8) for subsequent analyses [13,16]. The bacterial strains and media are detailed in Table S6.

### 3.2. Ethanol Extraction of Bacteriostatic Substances from the Rhizome of R. madaio

Fresh roots of *R. madaio* were harvested in the herbal medicine plantation base in Liandu District, Lishui City, Zhejiang Province, China, in October 2023. The mature roots were brown in color and about 20 cm in length, with a distinct aroma. The plant was identified according to the Flora of China (2004) and the National Compilation of Chinese Herbal Medicines (2014). Bacteriostatic activity-related substances in the fresh *R. madaio* root sample were extracted using different concentrations (55%, 75%, and 95%) of ethanol [61]. Briefly, the fresh root sample was thoroughly washed, dried, pre-frozen, and then freeze-dried at −80 °C for 48 h. The freeze-dried sample was pulverized into powder. Subsequently, an aliquot of 100 mL of the different concentrations of ethanol (Analytical grade) was individually mixed with the powdered sample (10 g) at a solid-to-liquid ratio of 1:10 (*m*/*v*), macerating for 24 h at room temperature away from light. Then, the mixture was used for sonication, filtration, and concentrated by rotary evaporation. The equipment and running parameters were the same as detailed recently (e.g., [13,16]). The obtained paste extract after rotary evaporation was dissolved with sterile ultrapure water [62] and prepared a stock solution of 10 mg/mL, and then diluted appropriately for the following assays. The extraction ratio was expressed as the percentage of the weight (g) of the obtained crude extract (*Rm*EE) after rotary evaporation to the weight (g) of the *R. madaio* root powder sample. The *Rm*EEs extracted by 55%, 75%, and 95% ethanol were designated as *Rm*EE (55% E), *Rm*EE (75% E), and *Rm*EE (95% E), respectively.

### 3.3. Antibacterial Activity, Growth Curve, and Time-Kill Curve Analysis

The susceptibility of the tested strains to *Rm*EEs (100 mg/mL) was examined through the disk diffusion method approved by the Clinical and Laboratory Standards Institute, the United States (CLSI, M100-S23, 2018) [63]. MICs of *Rm*EEs were measured through the broth microdilution method (CLSI, M100-S18, 2018) [64]. A MIC was defined as the minimum concentration of a specific antibacterial agent that can inhibit bacterial growth. Unless otherwise noted, *E. coli* ATCC 25922 was used as a quality control strain, and CN (10 μg/mL) and sterile deionized water were used as positive and blank controls, respectively [13,16].

For the growth curve analysis, the two representative strains *S. aureus* ATCC 25923 and *V. cholerae* GIM 1.449 (mid-LGP) were incubated in the TSB medium containing *Rm*EE-F2 (1 × MIC, 391 μg/mL or 1/2 × MIC, 195.5 μg/mL) at 37 °C for 24 h, respectively. Growth curves were determined using Automatic Growth Curve Analyzer (Bioscreen C, Growth Curves USA, Piscataway, NJ, USA) [13,16]. Unless otherwise noted, bacterial culture without *Rm*EE-F2 was used as a control.

For the time-kill curve analysis, the two representative strains *S. aureus* ATCC 25923 and *V. cholerae* GIM 1.449 (mid-LGP) were individually incubated in the TSB with *Rm*EE-F2 (1 × MIC or 1/2 × MIC) at 37 °C for 24 h. The bacterial culture at 2 h, 4 h, 6 h, 8 h, 12 h, and 24 h was individually collected, diluted, and spread on the TSB agar plates, and then incubated at 37 °C for 24 h for viable colony counting [65].

### 3.4. Prep-HPLC Analysis

The *Rm*EE (75% E) was further purified using an Ultra HPLC Sunfire C18 column (Waters, Milford, MA, USA) and a Waters 2707 autosampler (Waters, Milford, MA, USA). The equipment and running parameters were the same as detailed previously (e.g., [13,16]). The detection of purified components was implemented in the wavelength range from 200 nm to 600 nm using a photodiode array detector (PDA), and single peaks were collected. The obtained fraction 2 of *Rm*EE (75% E) was designated as *Rm*EE-F2 in the following analyses.

### 3.5. Bacterial Cell Surface Biophysical Parameter Assays

Bacterial CSH was determined as described previously [13,16]. Briefly, *S. aureus* ATCC 25923 and *V. cholerae* GIM 1.449 (mid-LGP) were incubated in the TSB with *Rm*EE-F2 (1 × MIC) for 2 h, 4 h, and 6 h, respectively. Then, each bacterial culture (1 mL) was mixed with the probe hexadecane (1 mL) (TCI Development Co., Ltd., Shanghai, China) for 5 min, and kept for 30 min at 37 °C. The OD_600_ values were measured using BioTek Synergy H1 Microplate Reader (BioTek Instruments Inc., Winooski, VT, USA). Meanwhile, CMF and ICMP of each bacterial culture were measured with 1,6-diphenyl-1,3,5,-hexatriene (DPH, Shanghai Jizhi Biochemical Technology Co., Ltd., Shanghai, China) and o-nitrophenyl-β-galactopyranoside (ONPG, Jiangsu CWBIO Biotechnology Co., Ltd., Nanjing, China) as a probe, respectively [13,16]. Of these, the bacterial ICMP was detected every 30 min for 5 h.

### 3.6. Nucleotide Acid and Protein Exudation and SEM Assays

As described above, the tested strains (mid-LGP) were incubated in the TSB with *Rm*EE-F2 (1 × MIC) for 2 h, 4 h, and 6 h, respectively. Then, each bacterial culture (1 mL) was collected for centrifugation (4 °C, 3500 rpm, 5 min), and OD_260_ values were examined for each supernatant [16]. Concentrations of extracellular proteins were determined using the Bradford Method Protein Concentration Determination kit (Sinopharm Group Co., Ltd., Shanghai, China) [16].

Meanwhile, each bacterial culture was collected and subjected to the SEM observation (Hitachi SU5000, Tokyo, Japan, 30 kV, × 35,000 and × 10,000) [13,16].

### 3.7. Illumina RNA Sequencing and Analysis

As described above, *S. aureus* ATCC 25923 and *V. cholerae* GIM 1.449 (mid-LGP) were treated with *Rm*EE-F2 (1 × MIC) for 6 h, respectively. Total RNA extraction, purification, analysis, and Illumina RNA sequencing were carried out by Shanghai Majorbio Bio-pharm Technology Co., Ltd. (Shanghai, China) through the Illumina HiSeq 2500 platform (Illumina, Santiago, CA, USA) [13]. DEGs and altered metabolic pathways in *S. aureus* ATCC 25923 and *V. cholerae* GIM 1.449 were calculated and analyzed [13,16].

To confirm RNA sequencing data, RT-qPCR was performed [13]. The designed primers (Table S7) of representative DEGs were synthesized by Shanghai Sangon Biological Engineering Technology and Service Co., Ltd., Shanghai, China.

To link the enriched pathways (e.g., β-lactam resistance) to phenotypic outcomes (e.g., MIC changes), *S. aureus* ATCC 25923 was cultured in the TSB at 37 °C until mid-LGP, and *Rm*EE-F2 (1 × MIC) was supplemented and continuously incubated at 37 °C for 6 h. After the incubation, *S. aureus* ATCC 25923 cells were sampled by centrifugation, and the cell pellet was washed with sterile 1 × phosphate-buffered saline (PBS, pH 7.0, Sangon, Shanghai, China) three times, and re-suspended in 1 × PBS to 1 × 10^6^ CFU/mL. Subsequently, the bacterial cells were subjected to the MIC determination with AMP, according to the CLSI guidelines (CLSI, M100-S18, 2018) [64].

### 3.8. Assessment of Antibacterial Effect of RmEE-F2 on Artificially Spiked Fish and Shrimp

We sampled fresh *C. auratus* and *P. vannamei* at an aquatic product market in Shanghai, China. The samples were thoroughly washed and skinned or shelled using a sterile scalpel and tweezers. The meat samples were cut into pieces (1 ± 0.1 g) and rinsed three times with sterile water before being UV sterilized for 20 min. Next, 1.0 g of each meat sample was homogenized with 9 mL of the sterile 1 × PBS for 3 min. Then, the homogenized mixture was directly spread onto TSB agar plates (100 μL/plate) and incubated at 37 °C overnight. Only if no colony on the plates was observed, then the meat samples could be used in the following analysis [66].

*S. aureus* ATCC 25923 and *V. cholerae* GIM 1.449 (mid-LGP, about 1 × 10^8^ CFU/mL) were inoculated into the meat samples, respectively. The following groups were set up: NG: 1 g of the meat sample + the bacterial culture (10 μL, final concentration of 1 × 10^6^ CFU/g); TG: 1 g of the meat sample + the bacterial culture (1 × 10^6^ CFU/g) + *Rm*EE-F2 solution (100 μL, 391 µg/mL); PG: 1 g of the meat sample + the bacterial culture (1 × 10^6^ CFU/g) + doxycycline (100 μL, 10 μg/mL) or vancomycin (100 μL, 10 μg/mL) solutions against *S. aureus* and *V. cholerae*, respectively. Blank control group: 1 g of the meat sample, as described above.

These groups were stored at 4 °C for 0 h, 6 h, 12 h, and 24 h, respectively. Subsequently, viable *S. aureus* ATCC 25923 and *V. cholerae* GIM 1.449 cells were counted as described above. Bactericidal rate was calculated as follows: Bactericidal rate (%) = (Ba − Bt)/Ba × 100%. Ba: bacterial count of the NG (CFU/g); and Bt: bacterial count of the TG (CFU/g).

### 3.9. Assessment of Quality and Sensory of the Fish and Shrimp Treated by RmEE-F2

The quality and sensory properties of the fish and shrimp samples before and after treatment by *Rm*EE-F2 were assessed as described recently [67]. Briefly, aliquots of *P. vannamei and C. auratus* meat samples were immersed in the *Rm*EE-F2 solution (391 μg/mL, 1:1, *m*/*v*) for 30 min, respectively. The commonly used chemical preservative potassium sorbate (2.5%, *w*/*v*, Shanghai Wechem Chemical Co., Ltd., Shanghai, China) was used as a positive control, and the untreated samples as negative controls. All the samples were stored in sterile plastic sampling bags (Shiray Scientific Trading Co., Ltd., Shanghai, China) at 4 °C for 8 days. During the storage, aliquots of the samples were subjected to the following assays on day 2, day 4, day 6, and day 8, respectively.

For the pH assay, an aliquot (2 g) of the samples was mixed with 18 mL of sterile deionized water and homogenized at 5000 rpm for 2 min. After filtering to delete the minced meat residue, pH values of the filtrate were examined using a digital pH meter [68].

For the TBARS assay, briefly, an aliquot (10 g) of the samples was homogenized with trichloroacetic acid solution (25 mL, 10%, *v*/*v*) at 1000 rpm for 30 s. After filtration, the sample (5 mL) was mixed with 0.02 M thiobarbituric acid (TBA, 5 mL, Shanghai Macklin Biochemical Co., Ltd., Shanghai, China) and incubated at 80 ± 2 °C for 35 min. After cooling to room temperature, OD_532_ values of the samples were examined. According to the standard curve prepared using MDA (Shanghai Macklin Biochemical Co., Ltd., Shanghai, China), TBARS values were calculated and expressed in milligrams of MDA per kilogram of the meat sample [68].

For the TVB-N assay, briefly, an aliquot (3 g) of the meat sample and 1 g of magnesium oxide were added to a digestion tube, and then 0.1 M hydrochloric acid was added as the titrant. Quantitative analysis was performed using the Kjeltec 8400 Automatic Kjeldahl Nitrogen Determination apparatus (FOSS Analytical, Hillerød, Denmark). The data were expressed as TVB-N mg per 100 g of the meat sample [69].

For the sensory evaluation, based on the criteria described recently by Jumilla-Lorenz et al. [70], a sensory assessment group was organized, including eight trained college students (5 male and 3 female) in the specialty of food science and technology, in a blinded manner by the panelists to ensure accuracy and minimize subjective bias in the assessment scores. The sensory assessment scores are shown in Table S8, including the surface condition, texture, firmness, odor, and overall color of the *C. auratus* and *P. vannamei* meat samples. The scores ranged from 1 to 5, and scores below 3 were rejected, and scores above 3 were accepted.

### 3.10. Cytotoxicity Assay

Cytotoxicity of *Rm*EE-F2 on human Caco-2, A549, and HepG-2 cell lines (Gibco, Thermo Fisher Scientific, Waltham, MA, USA) was evaluated using the CCK-8 kit (Sigma-Aldrich, Saint Louis, MO, USA) [71]. The Caco-2 cell line is commonly used to study the transport, absorption, and permeability of substances, including drugs, in the human gut [72]. Moreover, *V. cholerae* can cause severe watery diarrhea [73]. The HepG-2 cell line is often used for the drug-induced hepatotoxicity assessment [74], while the A549 cell line can mimic the physiological and pathological status of the human lung. The Caco-2, A549, and HepG-2 cells (1 × 10^4^ cells/well) were homogeneously dispersed in sterile 96-well plates, which were added with 100 µL/well of complete medium supplemented with 10% fetal bovine serum FBS (*v*/*v*), 1% antibiotic (*v*/*v*, 100 × penicillin-streptomycin concentrate; Gibco, Waltham, MA, USA). The former two cells were incubated in Dulbecco’s modified eagle medium (DMEM), while the latter in Ham’s F12K medium at 37 °C (5% CO_2_) for 24 h. Then, the cells were gently washed using the sterile 1 × PBS buffer (Gibco, Waltham, MA, USA) for 2–3 times. *Rm*EE-F2 was diluted with the complete medium to the concentrations of 1563, 781, 391, 195, 98, and 49 µg/mL, respectively. The tested cells were continuously incubated in the medium supplemented with each *Rm*EE-F2 solution for 24 h. Then, CCK8 (10 µL/well) was added and incubated for 4 h, and the OD_450_ value of each well was measured. Cell viability was calculated: Cell viability (%) = (V_1_ − V_0_)/(V_2_ − V_0_) × 100%. V_1_: absorbance of the cell culture + *Rm*EE-F2 + CCK-8 solution; V_2_: absorbance of the cell culture + CCK-8 solution; V_0_: blank absorbance of the cell culture medium without the cells + CCK-8 solution.

### 3.11. Caco-2 Cell Infection Assay

Caco-2 cell infection by the tested strains was performed in vitro [75]. The following groups were set up: NG: Caco-2 cells without bacterial infection and treatment; MG: Caco-2 cells infected with *V. cholerae* GIM 1.449 or *S. aureus* ATCC 25923 (bacteria-to-cell ratio, MOI = 30); TG: Caco-2 cells infected with *V. cholerae* GIM 1.449 or *S. aureus* ATCC 25923, and pre-treated with *Rm*EE-F2 solution (1 × MIC, 391 µg/mL); PG: Caco-2 cells infected with *V. cholerae* GIM 1.449 or *S. aureus* ATCC 25923, and pre-treated with cefepime (4 µg/mL) (Macklin Biochemical Technology Co., Ltd., Shanghai, China). These groups were cultured at 37 °C (5% CO_2_) for 24 h, and Caco-2 cell viability was determined as described above.

### 3.12. Ultra HPLC-Mass Spectrometry (UHPLC-MS) Analysis

The *Rm*EE-F2 was diluted with sterile ultrapure water (analytical grade), and the diluted sample (10 mg/mL, 1 mL) was subjected to UHPLC-MS analysis by Shanghai Baiqu Biological Co., Ltd. (Shanghai, China). The UHPLC-MS was performed with the same parameters described previously [13,16].

### 3.13. Data Analysis

The SPSS version 17.0 software (SPSS Inc., Armonk, NY, USA) was utilized to analyze the data through one-way analysis of variance (ANOVA) followed by an appropriate post-hoc test (Tukey), with a uniform significance level of α = 0.05. All of the tests were performed in independent biological triplicate, and the final data were presented as mean ± SD of three triplicate.

## 4. Conclusions

In this study, ethanol was employed as a solvent to extract antibacterial components from the rhizome of *R. madaio*. The extraction ratios of the sample were 25.57%, 33.8%, and 31.10% when 95%, 75%, and 55% ethanol were used, respectively. All the *Rm*EEs could inhibit 7 of the 9 tested bacterial species, but showed various DIZs (17.00–8.00 mm). The most pronounced inhibitory activity was manifested by *Rm*EE (95% E and 75% E) on Gram-positive *S. aureus* ATCC 25923, followed by Gram-negative *V. cholerae* GIM 1.449, showing DIZ values of 17.00 ± 0.50 and 11.50 ± 0.32 mm, respectively. Correspondingly, MIC values of 98 μg/mL and 391 μg/mL, respectively.

The purified *Rm*EE-F2 exhibited inhibition activities on *S. aureus* ATCC 25923 and *V. cholerae* GIM 1.449, with MIC values of 391 μg/mL. *Rm*EE-F2 (1 × MIC) significantly reduced CSH, but increased CMF and ICMP of *S. aureus* ATCC 25923 and *V. cholerae* GIM 1.449. *Rm*EE-F2 markedly eliminated the two tested strains in the spiked *C*. *auratus* and *P*. *vannamei* samples, and manifested bacteriostatic rates of 88.21–91.17% after being treated at 4 °C for 24 h. Meanwhile, *Rm*EE-F2 effectively rendered the pH rising and reduced lipid oxidation and protein degradation of the *C. auratus* and *P. vannamei* samples during the low-temperature preservation for 6 days, extending the shelf life of the aquatic products.

*Rm*EE-F2 (<781 µg/mL) showed non-cytotoxicity to human colon Caco-2, liver HepG-2, and lung A549 cell lines, and rescued *V. cholerae* and *S. aureus*-infected Caco-2 cells by enhancing viability of 14.31–16.60% (1 × MIC).

Comparative transcriptome analysis uncovered that *Rm*EE-F2 mainly inhibited protein synthesis and β-lactam resistance, resulting in impaired growth, the elimination of drug resistance, and even death of *S. aureus* ATCC 25923; and mainly repressed DNA replication and repair, cell wall and membrane synthesis, and protein synthesis, resulting in cell damage and death of *V. cholerae* GIM 1.449. The major potential antibacterial compounds in *Rm*EE-F2 included melibiose (9.86%), 3-Nid (7.12%), and citric acid (6.07%).

Overall, this study unlocks the potential of *Rm*EE-F2 as a natural food preserver for fighting the pathogens in aquatic products, paving the way for further research on the in vivo antibacterial activity of *Rm*EE-F2.

## Figures and Tables

**Table 1:** The top 10 potential antibacterial compounds identified in *Rm*EE-F2 by UHPLC-MS analysis.

Identified Compound	Compound Nature	Rt (min)	Formula	Exact Mass	Area (%)
Melibiose	Carbohydrates	25.5	C_12_H_22_O_11_	365.1040	9.86
3-(N, N-dimethylaminomethyl)indole	Alkaloids	26.4	C_11_H_14_N_2_	175.1183	7.12
Citric Acid	Fatty acids	27.5	C_6_H_8_O_7_	191.0195	6.01
3-Methylbutanamine	Alkaloids	356.5	C_5_H_13_N	88.1118	5.18
Otonecine	Alkaloids	51.2	C_9_H_15_NO_3_	186.1118	4.42
Pongamol	Flavonoids	30.1	C_18_H_14_O_4_	317.0830	4.29
Ovalitenin B	Flavonoids	30.8	C_19_H_18_O_4_	309.1188	2.97
Isorhapontin	Stilbenoids	206.8	C_21_H_24_O_9_	419.1342	2.93
Sucrose	Carbohydrates	26.0	C_12_H_22_O_11_	341.1084	2.35
Turanose	Carbohydrates	26.0	C_12_H_22_O_11_	341.1084	2.35
Palatinose (hydrate)	Carbohydrates	26.0	C_12_H_22_O_11_	341.1084	2.35

## Data Availability

A complete list of DEGs in the two strains was available in the NCBI SRA database (https://www.ncbi.nlm.nih.gov/sra/, accessed on 25 November 2024) under the accession number PRJNA1187379.

## Supplementary Materials

The following supporting information can be downloaded at: https://www.mdpi.com/article/10.3390/foods14203479/s1, Table S1: The antibacterial activities of *Rm*EEs; Table S2: The antibacterial activities of the purified fractions from *Rm*EE (75% E) through Pre-HPLC; Table S3: The major altered metabolic pathways in *S. aureus* ATCC25923 triggered by *Rm*EE-F2; Table S4: The major altered metabolic pathways in *V. cholerae* GIM1.449 triggered by *Rm*EE-F2; Table S5: The compounds identified in *Rm*EE-F2 by UHPLC-MS analysis; Table S6: The bacterial strains and media used in this study; Table S7: The primers designed and used in the RT-qPCR assay; Table S8: The sensory assessment items of the fish and shrimp meat samples; Figure S1: Relative expression levels of the representative DEGs by the RT-PCR assay.
