# Supplementary material for: Antibacterial Efficacy of Ethanol Extracts from Edible Rumex madaio Root and Application Potential for Eliminating Staphylococcus aureus and Vibrio cholerae in Aquatic Products for Green Food Preservation
Source: Foods. 2025 Oct 12;14(20):3479. doi: 10.3390/foods14203479 (PMC12564717; doi:10.3390/foods14203479)
Supplement: Supplementary file 1 [file foods-14-03479-s001.zip › foods-3855319-supplementary.pdf]

Supplementary files

Table S1. The antibacterial activities of *Rm*EEs.

**Bacterial Strain**	**DIZ (mm)**				**MIC (μg/mL)**		
	***Rm*EE (95% E)**	***Rm*EE (75% E)**	***Rm*EE (55% E)**	**CN**	***Rm*EE (95% E)**	***Rm*EE (75% E)**	***Rm*EE (55% E)**
A. hydrophila ATCC 35654	10.00 ± 0.25	10.50 ± 0.26	8.50 ± 0.32	19 ± 0.25	1563	1563	3125
B. cereus Y 1	15.50 ± 0.34	15.50 ± 0.23	12.50 ± 0.25	19 ± 0.20	391	391	781
E. cloacae ATCC 13047	–	–	–	15 ± 0.35	–	–	–
E. coli ATCC 25922	–	–	–	17 ± 0.50	–	–	–
S. dysenteriae CMCC 51252	11.00 ± 0.26	10.50 ± 0.36	9.00 ± 0.25	22 ± 0.25	195	195	3125
S. aureus ATCC 25923	17.00 ± 0.50	16.50 ± 0.37	14.00 ± 0.42	18 ± 0.30	98	98	195
V. cholerae GIM 1.449	10.00 ± 0.34	11.50 ± 0.32	9.00 ± 0.29	23 ± 0.20	391	391	781
V. metschnikovii ATCC 700040	10.00 ± 0.23	10.50 ± 0.28	10.00 ± 0.24	22 ± 0.25	781	781	781
V. parahaemolyticus ATCC 17802	10.00 ± 0.21	13.00 ± 0.54	8.00 ± 0.23	18 ± 0.25	391	391	1563

Note: *Rm*EEs: different concentrations of ethanol extracts of *R. madaio* root. –: no antibacterial activity. DIZ: diameters of inhibitory zone, including the diameter of the disc (6 mm). MIC: minimum inhibitory concentration. CN: gentamicin (10 μg/mL). The values are expressed as the mean ± standard deviation (S.D.) of three parallel measurements.

Table S2. The antibacterial activities of the purified fractions from *Rm*EE (75 % E) through Pre-HPLC.

**Tested strains**	**DIZ (mm)**			**MIC (μg/mL)**		
	***Rm*EE-F1**	***Rm*EE-F2**	***Rm*EE-F3**	***Rm*EE-F1**	***Rm*EE-F2**	***Rm*EE-F3**
A. hydrophila ATCC 35654	–	8.00 ± 0.25	–	–	1563	–
B. cereus Y 1	–	10.50 ± 0.25	9.50 ± 0.5	–	781	781
E. sakazakii CMCC 45401	–	7.00 ± 0.40	–	–	1563	–
S. aureus ATCC 25923	8.00 ± 0.5	11.50 ± 0.50	–	1563	391	–
S. dysenteriae CMCC 51252	–	9.00 ± 0.15	–	–	781	–
V. cholerae GIM 1.449	–	10.50 ± 0.35	–	–	391	–
V. metschnikovii ATCC 700040	–	8.50 ± 0.25	–	–	781	–
V. parahaemolyticus ATCC 17802	8.00 ± 0.25	10.00 ± 0.50	–	1563	781	–

Note: *Rm*EE-F1 to *Rm*EE-F3: The purified fractions from *Rm*EE (75 % E) through Pre-HPLC. –: no antibacterial activity. DIZ: diameters of inhibitory zone, including the diameter of the disc (6 mm). MIC: minimum inhibitory. The values are expressed as the mean ± standard deviation (S.D.) of three parallel measurements.

Table S3. The major altered metabolic pathways in *S. aureus* ATCC25923.

**Metabolic Pathway**	**Gene_ID**	**Fold Change**	**Gene Description**
Valine, leucine and isoleucine biosynthesis			
	PQQ26_11140	0.305	Acetolactate synthase AlsS
	PQQ26_10305	0.268	3-isopropylmalate dehydrogenase
	PQQ26_10300	0.278	2-isopropylmalate synthase
	PQQ26_10320	0.225	Threonine ammonia-lyase IlvA
	PQQ26_10295	0.334	Ketol-acid reductoisomerase
	PQQ26_10285	0.362	Biosynthetic-type acetolactate synthase large subunit
	PQQ26_10315	0.230	3-isopropylmalate dehydratase small subunit
	PQQ26_10280	0.450	Dihydroxy-acid dehydratase
	PQQ26_06895	0.060	Bifunctional threonine ammonia-lyase/L-serine ammonia-lyase TdcB
	PQQ26_10290	0.421	ACT domain-containing protein
	PQQ26_04320	4.808	LeuA family protein
Riboflavin metabolism			
	PQQ26_08910	10.061	Bifunctional diaminohydroxyphosphoribosylaminopyrimidine deaminase
	PQQ26_08900	10.789	3%2C4-dihydroxy-2-butanone-4-phosphate synthase
	PQQ26_08905	10.757	Riboflavin synthase
	PQQ26_04295	3.408	Cof-type HAD-IIB family hydrolase
	PQQ26_08895	7.225	6%2C7-dimethyl-8-ribityllumazine synthase
	PQQ26_10515	2.887	Hydroxyethylthiazole kinase
	PQQ26_01765	3.965	NADPH-dependent oxidoreductase
	PQQ26_05955	2.016	Riboflavin biosynthesis protein RibF
	PQQ26_07550	2.817	NUDIX hydrolase
Ribosome metabolism			
	PQQ26_11240	0.348	50S ribosomal protein L15
	PQQ26_05790	0.299	50S ribosomal protein L19
	PQQ26_02565	0.240	50S ribosomal protein L7/L12
	PQQ26_07075	2.630	30S ribosomal protein S1
	PQQ26_02595	0.489	30S ribosomal protein S7
	PQQ26_11285	0.216	50S ribosomal protein L14
	PQQ26_11255	0.359	50S ribosomal protein L18
	PQQ26_02560	0.481	50S ribosomal protein L10

	PQQ26_02585	0.353	Ribosomal L7Ae/L30e/S12e/Gadd45 family protein
	PQQ26_11290	0.460	30S ribosomal protein S17
	PQQ26_05750	2.976	Ribonuclease III
	PQQ26_02535	2.425	50S ribosomal protein L33
	PQQ26_06330	3.134	Homoserine dehydrogenase
Lysine biosynthesis			
	PQQ26_08820	3.557	Mn(2+)-dependent dipeptidase Sapep
	PQQ26_06700	0.332	Diaminopimelate decarboxylase
	PQQ26_06665	0.453	Aspartate kinase
	PQQ26_06675	0.420	4-hydroxy-tetrahydrodipicolinate synthase
	PQQ26_06680	0.407	4-hydroxy-tetrahydrodipicolinate reductase
	PQQ26_06670	0.492	Aspartate-semialdehyde dehydrogenase
	PQQ26_10005	2.449	ArgE/DapE family deacylase
	PQQ26_06325	2.836	Aspartate kinase
C5-Branched dibasic acid metabolism			
	PQQ26_11140	0.305	Acetolactate synthase AlsS
	PQQ26_10305	0.268	3-isopropylmalate dehydrogenase
	PQQ26_10285	0.362	Biosynthetic-type acetolactate synthase large subunit
	PQQ26_11135	0.272	Acetolactate decarboxylase
	PQQ26_10315	0.230	3-isopropylmalate dehydratase small subunit
	PQQ26_10290	0.421	ACT domain-containing protein
Arginine biosynthesis			
	PQQ26_04235	0.094	Argininosuccinate lyase
	PQQ26_09860	0.415	Nitric oxide synthase oxygenase
	PQQ26_11540	5.246	Urease subunit alpha
	PQQ26_13330	16.226	Arginine deiminase
	PQQ26_10860	4.573	Arginase
	PQQ26_04240	0.193	Argininosuccinate synthase
	PQQ26_13325	6.525	Ornithine carbamoyltransferase
	PQQ26_05425	0.371	Carbamate kinase
	PQQ26_11535	18.991	Urease subunit beta
	PQQ26_00655	0.176	N-acetyl-gamma-glutamyl-phosphate reductase
	PQQ26_00645	0.240	Acetylglutamate kinase
	PQQ26_11530	12.02	Urease subunit gamma
Monobactam biosynthesis			
	PQQ26_06665	0.453	Aspartate kinase

	PQQ26_06675	0.420	4-hydroxy-tetrahydrodipicolinate synthase
	PQQ26_06680	0.407	4-hydroxy-tetrahydrodipicolinate reductase
	PQQ26_06670	0.492	Aspartate-semialdehyde dehydrogenase
	PQQ26_06325	2.836	Aspartate kinase
β-Lactam resistance			
	PQQ26_05490	0.329	Penicillin-binding protein
	PQQ26_03090	2.941	ABC transporter ATP-binding protein
	PQQ26_04385	0.156	Peptide ABC transporter substrate-binding protein
	PQQ26_04375	0.339	ABC transporter ATP-binding protein
	PQQ26_04380	0.323	ATP-binding cassette domain-containing protein
	PQQ26_04365	0.495	ABC transporter permease
	PQQ26_04370	0.483	ABC transporter permease
Thiamine metabolism			
	PQQ26_11230	0.340	Adenylate kinase
	PQQ26_10525	8.808	Thiaminase II
	PQQ26_10520	3.758	Bifunctional hydroxymethylpyrimidine kinase/phosphomethylpyrimidine kinase
	PQQ26_08175	2.879	Cysteine desulfurase family protein
	PQQ26_10515	2.887	Hydroxyethylthiazole kinase
	PQQ26_10510	2.507	Thiamine phosphate synthase
	PQQ26_08620	0.395	TRNA 4-thiouridine(8) synthase ThiI
	PQQ26_13285	4.364	Alkaline phosphatase

Table S4. The major altered metabolic pathways in *V. cholerae* GIM1.449.

**Metabolic Pathway**	**Gene_ID**	**Fold Change**	**Gene Description**
Mismatch repair			
	GTH07_10225	0.356	Exodeoxyribonuclease VII large subunit
	GTH07_11350	0.257	DNA mismatch repair protein MutS
	GTH07_08030	0.208	Exodeoxyribonuclease I
	GTH07_12235	0.316	DNA mismatch repair endonuclease MutL
	GTH07_01335	0.260	Dam family site-specific DNA-(adenine-N6)-methyltransferase
	GTH07_09650	0.209	Exodeoxyribonuclease VII small subunit
	GTH07_10680	0.315	DNA mismatch repair endonuclease MutH

	GTH07_11935	0.225	Single-stranded DNA-binding protein
	GTH07_03160	0.375	DNA polymerase III subunit alpha
	GTH07_00010	0.350	DNA polymerase III subunit beta
	GTH07_13050	0.351	DNA helicase II
	GTH07_03215	0.343	DNA polymerase III subunit epsilon
	GTH07_10740	0.195	DNA polymerase III subunit psi
	GTH07_09265	0.388	NAD-dependent DNA ligase LigA
	GTH07_07765	0.281	3'-5' exonuclease
	GTH07_04310	0.183	DNA polymerase III subunit delta'
	GTH07_09345	0.312	DNA polymerase III subunit delta
	GTH07_02375	0.469	Single-stranded-DNA-specific exonuclease RecJ
	GTH07_08890	0.468	DNA polymerase III subunit gamma/tau
	GTH07_01930	0.324	DNA polymerase III subunit chi
Aminoacyl-tRNA biosynthesis			
	GTH07_07740	0.322	Asparagine-tRNA ligase
	GTH07_09335	0.259	Leucine-tRNA ligase
	GTH07_09150	0.188	Glutamine-tRNA ligase
	GTH07_08350	0.277	Aspartate-tRNA ligase
	GTH07_08625	0.307	Serine-tRNA ligase
	GTH07_05095	0.278	Cysteine-tRNA ligase
	GTH07_14665	0.369	Threonine-tRNA ligase
	GTH07_11305	0.297	Alanine-tRNA ligase
	GTH07_10620	0.333	Isoleucine-tRNA ligase
	GTH07_10700	0.252	Lysine-tRNA ligase
	GTH07_01925	0.322	Valine-tRNA ligase
	GTH07_10875	0.220	Tyrosine-tRNA ligase
	GTH07_04020	0.221	Arginine-tRNA ligase
	GTH07_09720	0.293	Proline-tRNA ligase
	GTH07_00040	0.339	Glycine-tRNA ligase subunit beta
	GTH07_10255	0.300	Histidine-tRNA ligase
	GTH07_08080	0.348	Phenylalanine-tRNA ligase subunit alpha
	GTH07_00045	0.222	Glycine-tRNA ligase subunit alpha
	GTH07_08075	0.492	Phenylalanine-tRNA ligase subunit beta
	GTH07_01350	0.376	Tryptophan-tRNA ligase
	GTH07_08975	0.229	Methionine-tRNA ligase
	GTH07_03300	0.221	Glutamate-tRNA ligase

	GTH07_00160	0.382	Methionyl-tRNA formyltransferase
Purine metabolism			
	GTH07_00955	0.241	Bifunctional GTP diphosphokinase/guanosine-3'%2C5'-bis pyrophosphate 3'-pyrophosphohydrolase
	GTH07_09745	0.329	Phosphoribosylformylglycinamidine synthase
	GTH07_02205	0.413	GTP diphosphokinase
	GTH07_00195	0.319	5-(carboxyamino)imidazole ribonucleotide mutase
	GTH07_12510	0.409	Guanosine-5'-triphosphate-C3'-diphosphate diphosphatase
	GTH07_10420	0.342	Exopolyphosphatase
	GTH07_03245	0.234	Phosphoribosylformylglycinamidine cyclo-ligase
	GTH07_12645	0.296	Phosphoribosylamine--glycine ligase
	GTH07_16020	0.316	Gamma-glutamyltransferase family protein
	GTH07_00190	0.362	5-(carboxyamino)imidazole ribonucleotide synthase
	GTH07_08055	0.211	Phosphoribosylglycinamide formyltransferase 2
	GTH07_00895	0.377	ADP compounds hydrolase NudE
	GTH07_11720	0.472	Symmetrical bis(5'-nucleosyl)-tetraphosphatase
	GTH07_10215	0.253	Glutamine-hydrolyzing GMP synthase
	GTH07_08545	0.228	Adenylosuccinate lyase
	GTH07_03530	0.162	Ribose-phosphate pyrophosphokinase
	GTH07_10220	0.227	IMP dehydrogenase
	GTH07_03920	0.332	Phosphoglucomutase (alpha-D-glucose-1%2C6-bisphosphate-dependent)
	GTH07_13610	0.236	Purine-nucleoside phosphorylase
	GTH07_00525	0.233	Class I adenylate cyclase
	GTH07_01450	0.315	Adenylosuccinate synthase
	GTH07_01675	0.057	Sulfate adenylyltransferase subunit CysN
	GTH07_02285	0.200	ADP-ribose diphosphatase
	GTH07_15810	0.143	Anaerobic ribonucleoside-triphosphate reductase
	GTH07_11085	0.158	Hypoxanthine phosphoribosyltransferase
	GTH07_07935	0.304	Ribonucleoside-diphosphate reductase subunit alpha
	GTH07_09195	0.344	Adenylate kinase
	GTH07_01670	0.041	Sulfate adenylyltransferase subunit CysD
	GTH07_02295	0.394	3'%2C5'-cyclic-AMP phosphodiesterase
	GTH07_02380	0.310	Bifunctional metallophosphatase/5'-nucleotidase
	GTH07_17000	0.263	NUDIX hydrolase
	GTH07_02710	0.388	Purine-nucleoside phosphorylase
	GTH07_02210	0.328	Nucleoside triphosphate pyrophosphohydrolase

	GTH07_01660	0.417	2'%2C3'-cyclic-nucleotide 2'-phosphodiesterase
	GTH07_12640	0.332	Bifunctional phosphoribosylaminoimidazolecarboxamide Formyltransferase/IMP cyclohydrolase
	GTH07_01680	0.134	Adenylyl-sulfate kinase
	GTH07_00965	0.275	Guanylate kinase
	GTH07_03005	0.274	Xanthine phosphoribosyltransferase
	GTH07_10275	0.409	Nucleoside-diphosphate kinase
	GTH07_00750	0.214	Adenosine deaminase
	GTH07_17155	0.358	Inosine/guanosine kinase
	GTH07_09115	0.443	Amidophosphoribosyltransferase
	GTH07_08895	0.263	Adenine phosphoribosyltransferase
	GTH07_14250	0.369	GMP reductase
	GTH07_11605	0.395	XTP/dITP diphosphatase
	GTH07_17915	0.375	DUF1255 family protein
	GTH07_03240	0.420	Phosphoribosylglycinamide formyltransferase
	GTH07_10530	0.205	Non-canonical purine NTP phosphatase
	GTH07_04490	0.458	5'-deoxynucleotidase
Peptidoglycan biosynthesis			
	GTH07_09375	0.257	Serine hydrolase
	GTH07_02455	0.321	UDP-N-acetylmuramate--L-alanine ligase
	GTH07_11015	0.388	Penicillin-binding protein 1B
	GTH07_10870	0.276	Serine-type D-Ala-D-Ala carboxypeptidase
	GTH07_16115	0.166	D-alanine--D-alanine ligase
	GTH07_01290	0.344	Penicillin-binding protein 1A
	GTH07_09360	0.231	Penicillin-binding protein 2
	GTH07_02420	0.325	Penicillin-binding protein 3
	GTH07_01875	0.419	UDP-N-acetylglucosamine 1-carboxyvinyltransferase
	GTH07_12430	0.389	UDP-N-acetylmuramate dehydrogenase
	GTH07_02425	0.360	UDP-N-acetylmuramoyl-L-alanyl-D-glutamate--2%2C6-diaminopimelate ligase
	GTH07_03105	0.384	Di-trans%2Cpoly-cis-decaprenylcistransferase
	GTH07_11395	0.271	Undecaprenyl-diphosphate phosphatase
	GTH07_02430	0.412	UDP-N-acetylmuramoyl-tripeptide--D-alanyl-D-alanine ligase
	GTH07_02440	0.402	UDP-N-acetylmuramoyl-L-alanine--D-glutamate ligase
	GTH07_02435	0.373	Phospho-N-acetylmuramoyl-pentapeptide-transferase
	GTH07_02450	0.431	Undecaprenyldiphospho-muramoylpentapeptide beta-N-acetylglucosaminyltransferase
	GTH07_02030	0.441	Phosphatase PAP2 family protein

DNA replication			
	GTH07_11435	0.237	DNA primase
	GTH07_03210	0.239	Ribonuclease HI
	GTH07_03155	0.374	Ribonuclease HII
	GTH07_11935	0.225	Single-stranded DNA-binding protein
	GTH07_03160	0.375	DNA polymerase III subunit alpha
	GTH07_12110	0.282	Replicative DNA helicase
	GTH07_00010	0.350	DNA polymerase III subunit beta
	GTH07_03215	0.343	DNA polymerase III subunit epsilon
	GTH07_00470	0.442	DNA polymerase I
	GTH07_10740	0.195	DNA polymerase III subunit psi
	GTH07_09265	0.388	NAD-dependent DNA ligase LigA
	GTH07_07765	0.281	3'-5' exonuclease
	GTH07_04310	0.183	DNA polymerase III subunit delta'
	GTH07_09345	0.312	DNA polymerase III subunit delta
	GTH07_08890	0.468	DNA polymerase III subunit gamma/tau
	GTH07_01930	0.324	DNA polymerase III subunit chi
Fatty acid biosynthesis			
	GTH07_04270	0.177	Ketoacyl-ACP synthase III
	GTH07_06835	0.428	Bifunctional 3-hydroxydecanoyl-ACP dehydratase/trans-2-decenoyl-ACP isomerase
	GTH07_16925	0.378	Ketoacyl-ACP synthase III
	GTH07_03860	0.196	Beta-ketoacyl-ACP synthase I
	GTH07_04290	0.166	Beta-ketoacyl-ACP synthase II
	GTH07_04280	0.235	3-oxoacyl-ACP reductase FabG
	GTH07_02045	0.111	Long-chain fatty acid--CoA ligase
	GTH07_17085	0.090	Trans-2-enoyl-CoA reductase family protein
	GTH07_05605	0.319	Trans-2-enoyl-CoA reductase family protein
	GTH07_12550	0.260	Acetyl-CoA carboxylase biotin carboxylase subunit
	GTH07_04275	0.219	ACP S-malonyltransferase
	GTH07_09135	0.346	Acetyl-CoA carboxylase carboxyltransferase subunit beta
	GTH07_03165	0.392	Acetyl-CoA carboxylase carboxyl transferase subunit alpha
	GTH07_12545	0.184	Acetyl-CoA carboxylase biotin carboxyl carrier protein
	GTH07_03140	0.312	3-hydroxyacyl-ACP dehydratase FabZ
Ubiquinone and other terpenoid-quinone biosynthesis			
	GTH07_04515	0.220	1, 4-dihydroxy-2-naphthoyl-CoA synthase

	GTH07_04520	0.187	O-succinylbenzoate synthase
	GTH07_07930	0.290	Bifunctional 3-demethylubiquinone 3-O-methyltransferase/2-octaprenyl-6-hydroxy phenol methylase
	GTH07_02090	0.409	2-octaprenyl-6-methoxyphenyl hydroxylase
	GTH07_01125	0.252	1, 4-dihydroxy-2-naphthoate polyprenyltransferase
	GTH07_00370	0.470	Bifunctional demethylmenaquinone Methyltransferase/2-methoxy-6-polyprenyl-1%2C4-benzoquinol methylase UbiE
	GTH07_00430	0.263	Chorismate lyase
	GTH07_16155	0.215	Hotdog fold thioesterase
	GTH07_04525	0.209	O-succinylbenzoate-CoA ligase
	GTH07_02095	0.494	FAD-dependent 2-octaprenylphenol hydroxylase
		0.366	2-succinyl-5-enolpyruvyl-6-hydroxy-3-cyclohexene-1-carboxylic-acid synthase
	GTH07_00425	0.354	4-hydroxybenzoate octaprenyltransferase
	GTH07_04510	0.291	2-succinyl-6-hydroxy-2%2C4-cyclohexadiene-1-carboxylate synthase
	GTH07_09300	0.338	2-octaprenyl-3-methyl-6-methoxy-1%2C4-benzoquinol hydroxylase
	GTH07_03600	0.372	NAD(P)H:quinone oxidoreductase
	GTH07_04500	0.183	Isochorismate synthase MenF
	GTH07_10190	9.471	Isochorismate synthase MenF
Homologous recombination			
	GTH07_11315	0.370	Recombinase RecA
	GTH07_00950	0.352	ATP-dependent DNA helicase RecG
	GTH07_01100	0.243	Primosomal protein N'
	GTH07_00015	0.447	DNA replication/repair protein RecF
	GTH07_08880	0.452	Recombination protein RecR
	GTH07_02815	0.456	Exodeoxyribonuclease V subunit gamma
	GTH07_05110	0.410	Holliday junction branch migration DNA helicase RuvB
	GTH07_02820	0.480	Exodeoxyribonuclease V subunit beta
	GTH07_05105	0.497	Holliday junction branch migration protein RuvA
	GTH07_05050	0.198	Prepilin peptidase
	GTH07_11935	0.225	Single-stranded DNA-binding protein
	GTH07_03160	0.375	DNA polymerase III subunit alpha
	GTH07_00010	0.350	DNA polymerase III subunit beta
	GTH07_03215	0.343	DNA polymerase III subunit epsilon
	GTH07_00470	0.442	DNA polymerase I
	GTH07_10740	0.195	DNA polymerase III subunit psi
	GTH07_07765	0.281	3'-5' exonuclease

	GTH07_04310	0.183	DNA polymerase III subunit delta'
	GTH07_09345	0.312	DNA polymerase III subunit delta
	GTH07_02375	0.469	Single-stranded-DNA-specific exonuclease RecJ
	GTH07_08890	0.468	DNA polymerase III subunit gamma/tau
	GTH07_01930	0.324	DNA polymerase III subunit chi
Nucleotide excision repair			
	GTH07_11950	0.241	Excinuclease ABC subunit UvrA
	GTH07_04910	0.268	Transcription-repair coupling factor
	GTH07_09045	0.326	Excinuclease ABC subunit UvrB
	GTH07_08120	0.373	Excinuclease ABC subunit UvrC
	GTH07_13050	0.351	DNA helicase II
	GTH07_00470	0.442	DNA polymerase I
	GTH07_09265	0.388	NAD-dependent DNA ligase LigA
Monobactam biosynthesis			
	GTH07_17250	0.449	Aspartate kinase
	GTH07_01675	0.057	Sulfate adenylyltransferase subunit CysN
	GTH07_01670	0.041	Sulfate adenylyltransferase subunit CysD
	GTH07_03640	0.416	4-hydroxy-tetrahydrodipicolinate synthase
	GTH07_11965	0.313	Lysine-sensitive aspartokinase 3
	GTH07_11300	0.221	Aspartate kinase
	GTH07_04215	0.342	Aspartate-semialdehyde dehydrogenase
	GTH07_03870	0.196	Aspartate-semialdehyde dehydrogenase
	GTH07_02495	0.448	4-hydroxy-tetrahydrodipicolinate reductase
	GTH07_01075	0.449	Bifunctional aspartate kinase/homoserine dehydrogenase II

Table S5. The compounds identified in *Rm*EE-F2 by UHPLC-MS analysis.

**Identified Compound**	**Compound Nature**	**Rt (min)**	**Formula**	**Exact Mass**	**Area (%)**
Melibiose	Carbohydrates	25.5	$C_{12}H_{22}O_{11}$	365.1040	9.86
3-(N, N-dimethylaminomethyl)indole	Alkaloids	26.4	$C_{11}H_{14}N_2$	175.1183	7.12
Citric Acid	Fatty acids	27.5	$C_6H_8O_7$	191.0195	6.01
3-Methylbutanamine	Alkaloids	356.5	$C_5H_{13}N$	88.1118	5.18
Otonecine	Alkaloids	51.2	$C_9H_{15}NO_3$	186.1118	4.42
Pongamol	Flavonoids	30.1	$C_{18}H_{14}O_4$	317.0830	4.29

Ovalitenin B	Flavonoids	30.8	$C_{19}H_{18}O_4$	309.1188	2.97
Isorhapontin	Stilbenoids	206.8	$C_{21}H_{24}O_9$	419.1342	2.93
Sucrose	Carbohydrates	26.0	$C_{12}H_{22}O_{11}$	341.1084	2.35
Turanose	Carbohydrates	26.0	$C_{12}H_{22}O_{11}$	341.1084	2.35
Palatinose (hydrate)	Carbohydrates	26.0	$C_{12}H_{22}O_{11}$	341.1084	2.35
Emodin	Polyketides	261.8	$C_{15}H_{10}O_5$	269.0452	2.33
Procyanidin B2	Flavonoids	155.5	$C_{30}H_{26}O_{12}$	577.1350	1.94
1-(2,6-dihydroxyphenyl)-9-phenyl-nonan-1-one	Polyketides	325.7	$C_{21}H_{26}O_3$	325.1839	1.84
Triptophenolide	Terpenoids	317.7	$C_{20}H_{24}O_3$	311.1683	1.72
6-[(E)-2-(3,4-dihydroxyphenyl)vinyl]-4-methoxy-pyran-2-one	Styrylpyrones	235.8	$C_{14}H_{12}O_5$	259.0610	1.71
Sorbose	Carbohydrates	24.5	$C_6H_{12}O_6$	203.0519	1.66
Tagatose	Carbohydrates	24.5	$C_6H_{12}O_6$	203.0519	1.66
6-Deoxyfagomine	Alkaloids	28.6	$C_6H_{13}NO_2$	132.1014	1.59
Malic acid	Fatty acids	26.8	$C_4H_6O_5$	133.0141	1.41
1-[8-hydroxy-3-methyl-1-[(2S,3R,4S,5S,6R)-3,4,5-trihydroxy-6-(hydroxymethyl) tetrahydropyran-2-yl]oxy-2-naphthyl]ethanone	Polyketides	203.1	$C_{19}H_{22}O_8$	377.1237	1.25
O-Tyrosine	Aminoacids and derivatives	28.3	$C_9H_{11}NO_3$	182.0805	1.23
3-Amino-3-(4-hydroxyphenyl) propanoic acid	Amino acids	28.3	$C_9H_{11}NO_3$	182.0805	1.23
5-hydroxy-2-(4-hydroxyphenyl)-7-[(2S,3R,4S,5S,6R)-3,4,5-trihydroxy-6-(hydroxymethyl)tetrahydropyran-2-yl]oxy-chroman-4-one	Flavonoids	198.3	$C_{21}H_{22}O_{10}$	433.1139	1.22
Procyanidin B1	Flavonoids	155.5	$C_{30}H_{26}O_{12}$	579.1477	1.12
Choline	Fatty acids	24.3	$C_5H_{14}NO$	104.1067	1.11
Oleamide	Fatty acids	308.0	$C_{18}H_{33}NO$	282.2781	1.03
Vasicinol	Alkaloids	70.3	$C_{11}H_{12}N_2O_2$	205.0965	0.96
Malonic acid	Fatty acids	26.8	$C_3H_4O_4$	103.0036	0.86
3-Hydroxypyruvic acid	Fatty acids	26.8	$C_3H_4O_4$	103.0036	0.86
Kaempferol	Flavonoids	226.4	$C_{15}H_{10}O_7$	285.0403	0.83
6-[(2E)-3,7-dimethylocta-2,6-dienyl]-7-hy	Coumarins	309.4	$C_{19}H_{22}O_3$	297.1527	0.82

droxy-chromen-2-one					
8-[(2E)-3,7-dimethylocta-2,6-dienyl]-7-hydroxy-chromen-2-one	Coumarins	309.4	$C_{19}H_{22}O_3$	297.1527	0.82
Cacticin	Flavonoids	145.8	$C_{22}H_{22}O_{12}$	477.1036	0.78
6-methoxy-4-methyl-chromen-2-one	Coumarins	202.6	$C_{11}H_{10}O_3$	191.0696	0.73
7-Methoxy-4-methylcoumarin	Coumarins	202.6	$C_{11}H_{10}O_3$	191.0696	0.73
Kaempferide_3-rhamnoside	Flavonoids	206.8	$C_{22}H_{22}O_{10}$	447.1272	0.72
1-[1,6-dihydroxy-3-methyl-8-[(2S,3R,4S,5S,6R)-3,4,5-trihydroxy-6-(hydroxymethyl)tetrahydropyran-2-yl]oxy-2-naphthyl]ethanone	Polyketides	148.2	$C_{19}H_{22}O_9$	393.1188	0.68
kaempferol-7-O-deoxyhexoside	Flavonoids	158.7	$C_{21}H_{20}O_{10}$	431.0876	0.67
(-)-Epicatechingallate	Flavonoids	176	$C_{22}H_{18}O_{10}$	441.0826	0.64
methyl asterrate	Polyketides	64.6	$C_{18}H_{18}O_8$	385.0911	0.60
5,7-dihydroxy-3-[(2S,3R,4S,5R,6R)-3,4,5-trihydroxy-6-(hydroxymethyl)oxan-2-yl]oxy-2-(3,4,5-trihydroxyphenyl)chromen-4-one	Flavonoids	152.7	$C_{21}H_{20}O_{13}$	479.0849	0.58
3-(3-Hydroxyphenyl)propanoic acid	Organic acid	156.7	$C_9H_{10}O_3$	165.0556	0.47
Desaminotyrosine	Organic acid	156.7	$C_9H_{10}O_3$	165.0556	0.47
2-Phenyllactic acid	Organic acid	156.7	$C_9H_{10}O_3$	165.0556	0.47
3-(2-Hydroxyphenyl)propanoic acid	Organic acid	156.7	$C_9H_{10}O_3$	165.0556	0.47
3-Methoxyphenylacetic acid	Organic acid	156.7	$C_9H_{10}O_3$	165.0556	0.47
1-Methylpyrrolidine	Alkaloids	28.6	$C_5H_{11}N$	86.09610	0.46
Anthraflavic_acid	Polyketides	158.1	$C_{14}H_8O_4$	523.1092	0.45
Procyanidin C1	Flavonoids	160.8	$C_{45}H_{38}O_{18}$	865.1970	0.45
Cinnamtannin B-1	Flavonoids	158.7	$C_{45}H_{36}O_{18}$	863.1829	0.44
5,13-bis(3,4-dihydroxyphenyl)-7-[2-(3,4-dihydroxyphenyl)-3,5,7-trihydroxy-chroman-8-yl]-4,12,14-trioxapentacyclo[11.7.1.02,11.03,8.015,20]henicosa-2(11),3(8),9,15,17,19-hexaene-6,9,17,19,21-pentol	Flavonoids	158.7	$C_{45}H_{36}O_{18}$	863.1829	0.44
Linoleamide	Fatty acids	297.4	$C_{18}H_{33}NO$	280.2626	0.41
P-Toluquinone	Polyketides	194.0	$C_7H_6O_2$	121.0295	0.41
Benzoic acid	Organic acid	194.0	$C_7H_6O_2$	121.0295	0.41
Tropolone	Organic acid	194.0	$C_7H_6O_2$	121.0295	0.41

Ent-Epicatechin-(4alpha-.8)-ent-epicatechin_3-gallate	Flavonoids	163.6	$C_{37}H_{30}O_{16}$	731.1585	0.40
3-methoxy-4-[3,4,5-trihydroxy-6-(hydroxymethyl)tetrahydropyran-2-yl]oxy-benzoic acid	Organic acid	55.9	$C_{14}H_{18}O_{9}$	329.0877	0.39
Eriodictyol	Flavonoids	140.6	$C_{15}H_{12}O_{6}$	289.0698	0.39
DEHP	Organic acid	321.0	$C_{24}H_{38}O_{4}$	391.2831	0.39
Tryptophan	Aminoacids and derivatives	69.7	$C_{11}H_{12}N_{2}O_{2}$	203.0825	0.38
Methyl pentadecanoate	Fatty acids	319.6	$C_{16}H_{32}O_{2}$	255.2328	0.37
Palmitic acid	Fatty acids	319.6	$C_{16}H_{32}O_{3}$	255.2328	0.37
2-[[7-hydroxy-1-(4-hydroxy-3,5-dimethoxyphenyl)-3-(hydroxymethyl)-6,8-dimethoxy-1,2,3,4-tetrahydronaphthalen-2-yl]methoxy]-6-(hydroxymethyl)oxane-3,4,5-triol	Lignans	69.7	$C_{28}H_{38}O_{13}$	203.0825	0.36
3,5-dihydroxy-2-(4-hydroxyphenyl)-7-[3,4,5-trihydroxy-6-(hydroxymethyl)oxan-2-yl]oxy-2,3-dihydrochromen-4-one	Flavonoids	319.6	$C_{21}H_{22}O_{11}$	255.2328	0.36
Mesaconic acid	Fatty acids	27.5	$C_{5}H_{6}O_{4}$	111.0087	0.36
3-methylolphenol	Organic acid	139.9	$C_{7}H_{8}O_{2}$	125.0594	0.34
4,7-dihydroxychromen-2-one	Coumarins	144.7	$C_{9}H_{6}O_{4}$	179.0334	0.33
Lansoprazole	Alkaloids	89.0	$C_{16}H_{14}F_{3}N_{3}O_{2}S$	370.0820	0.33
[(1S)-5-hydroxy-1-[(2S,3R,4S,5S,6R)-3,4,5-trihydroxy-6-(hydroxymethyl)oxan-2-yl]oxy-1,4a,5,7a-tetrahydrocyclopenta[c]pyran-7-yl]methyl benzoate	Terpenoids	208.8	$C_{22}H_{26}O_{10}$	449.1449	0.32
Lactate	Fatty acids	27.6	$C_{3}H_{6}O_{3}$	89.02430	0.31
3-Hydroxypropionic acid (beta-lactic acid)	Fatty acids	27.6	$C_{3}H_{6}O_{3}$	89.02430	0.31
4-ethoxychromen-2-one	Coumarins	202.6	$C_{11}H_{10}O_{3}$	189.0556	0.31
4-Aminobutyric acid (GABA)	Aminoacids and derivatives	26.6	$C_{4}H_{9}NO_{2}$	104.0702	0.30
(4S)-8-hydroxy-6-methoxy-4,5-dimethyl-3-methylene-isochroman-1-one	Coumarins	191.7	$C_{13}H_{14}O_{4}$	235.0957	0.30
2-methyl-3-[(2S,3R,4S,5S,6R)-3,4,5-trihy	Polyketides	49.2	$C_{12}H_{16}O_{8}$	289.0908	0.29

droxy-6-(hydroxymethyl)tetrahydropyran-2-yl]oxy-pyran-4-one					
Palmitamide	Fatty acids	305.6	$C_{16}H_{33}NO$	256.2627	0.29
(E)-3-(3,4-dihydroxyphenyl)-1-[2-hydroxy-4-[(2S,3R,4S,5S,6R)-3,4,5-trihydroxy-6-(hydroxymethyl)tetrahydropyran-2-yl]oxy-phenyl]prop-2-en-1-one	Flavonoids	197.8	$C_{21}H_{22}O_{10}$	435.1274	0.28
Methyl 8-hydroxy-9-oxo-xanthene-1-carboxylate	Polyketides	232.5	$C_{15}H_{10}O_5$	269.0455	0.28
Levonordefrin	Alkaloids	38.2	$C_9H_{13}NO_3$	166.0857	0.27
Normetanephrine	Alkaloids	38.2	$C_9H_{13}NO_3$	166.0857	0.27
10-methoxy-2,2-dimethyl-pyrano[3,2-g]chromen-8-one	Coumarins	206.4	$C_{15}H_{14}O_4$	259.0956	0.27

Rt: retention time

Table S6. The bacterial strains and media used in this study.

Bacterial Strain	Culture Medium	Source
Aeromonas hydrophila ATCC 35654	TSB	ATCC, USA
Bacillus cereus Y-1	TSB	Laboratory collection
Enterobacter cloacae ATCC 13047	TSB	ATCC, USA
Escherichia coli ATCC 25922	LB	ATCC, USA
Shigella dysenteriae CMCC 51252	TSB	IM, GAS, China
Staphylococcus aureus ATCC 25923	TSB	ATCC, USA
Vibrio cholerae GIM1.449	TSB	IM, GAS, China
Vibrio metschnikovii ATCC 700040	Marine 2216	ATCC, USA
Vibrio parahaemolyticus ATCC 17802	TSB	ATCC, USA

Note: ATCC, American Type Culture Collection; IM, GAS, Institute of Microbiology, Guangdong Academy of Sciences.

Table S7. The primers designed and used in the RT-qPCR assay.

Tested Strain	Target Gene	Primer Sequence (5' to 3')	Predicted Size (bp)
	16s RNA	GACACGGTCCAGACTCCTAC<br>GGTGCTTCTTCTGTCGCTAAC	179

S. aureus ATCC 25923	*argC*	CACCACATCTCGTACCAAT<br>GGCTGATTAGCATAATAAGAGG	146
	pbpA	GCAGTCCTACTTGTTGGTT<br>CCTCGTTCTGGTTGTTGT	155
	oppA	ACGCAGTTATTACAGCAGAT<br>TCGGAAGGAATTGAACCATT	208
	ilvA	CAAGCAATAGTAGCAGAACC<br>TACCACCACTAATGACACAA	217
	argG	CGTAAGTGGTGATGTCAGA<br>AACAGCAGCGTCTTGATT	136
V. cholerae GIM 1.449	*mrdA*	GGACAAGGCTATTGGACTG<br>GGTAAGTGGTGTACTCGGTCT	157
	fabF	GGTTGGCTTCGGTATGTC<br>ACTTGCTTAGTCCCTGCTTC	219
	mrcA	TGGGTGAAGCAGGGACTA<br>TCTCAACTTTACGGGCAAC	204
	murA	CAAAGAATGCCGCCCTAC<br>CGCCACCAATGGACCTAA	245
	bacA	CGGCACGGACAGCATTAC<br>TCAGCACCCGCTTCTTCA	192

Table S8. The sensory assessment items of the fish and shrimp meat samples.

Score	Odor	Overall Color	Surface Condition	Texture and Firmness
5 (Excellent)	Fish and shrimp smell is rich, no odor	White	The surface is moist without mucus	The muscle tissue is dense and has resilience when bent
4 (Good)	The smell is acceptable, there may be a slight ammonia odor	Yellowish white	The surface is slightly moist with a small amount of mucus	The muscles have a certain degree of elasticity, but the rebound is slightly slow.
3 (Fair)	A faint sour taste has appeared	Yellowish	The mucus has increased and slightly turbid.	The muscle tissue is loose and has poor resilience.
2 (Poor)	The putrid smell is obvious and pungent.	Grayish yellow	A large amount of mucus has increased, with an unusual odor	The muscle has softened and lost its elasticity.
1 (Very poor)	Very fishy and smelly	Gray	The mucus emits a foul and pungent odor	The muscle is completely loose and has lost its original shape

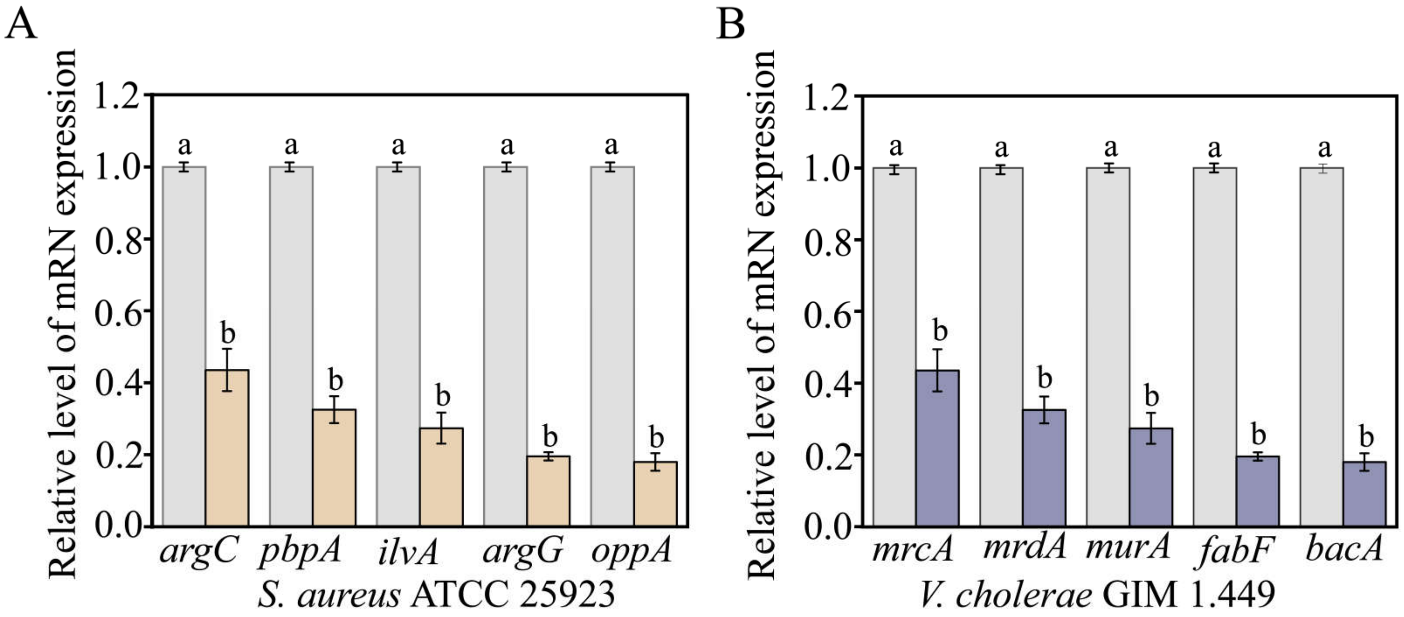

Figure S1. Relative expression levels of the representative DEGs by the RT-PCR assay.
